# Bidirectional associations between hearing difficulty and cognitive function in Chinese adults: a longitudinal study

**DOI:** 10.3389/fnagi.2023.1306154

**Published:** 2023-12-13

**Authors:** Xiaoyang Li, Mingyue Hu, Yinan Zhao, Ruotong Peng, Yongzhen Guo, Chi Zhang, Jundan Huang, Hui Feng, Mei Sun

**Affiliations:** Xiangya School of Nursing, Central South University, Changsha, Hunan, China

**Keywords:** reciprocal association, depressive symptoms, cognition, hearing, cross-lagged panel model

## Abstract

**Background:**

Middle-aged and older adults frequently experience hearing loss and a decline in cognitive function. Although an association between hearing difficulty and cognitive function has been demonstrated, its temporal sequence remains unclear. Therefore, we investigated whether there are bidirectional relationships between hearing difficulty and cognitive function and explored the mediating role of depressive symptoms in this relationship.

**Method:**

We used the cross-lagged panel model and the random-intercept cross-lagged panel model to look for any possible two-way link between self-reported hearing difficulty and cognitive function. To investigate depressive symptoms’ role in this association, a mediation analysis was conducted. The sample was made up of 4,363 adults aged 45 and above from the China Health and Retirement Longitudinal Study (CHARLS; 2011–2018; 44.83% were women; mean age was 56.16 years). One question was used to determine whether someone had a hearing impairment. The tests of cognitive function included episodic memory and intelligence. The Center for Epidemiologic Studies Depression Scale, which consists of 10 items, was used to measure depressive symptoms.

**Results:**

A bidirectional association between hearing and cognition was observed, with cognition predominating (Wald *χ*2 (1) = 7.241, *p* < 0.01). At the between-person level, after controlling for potential confounders, worse hearing in 2011 predicted worse cognitive function in 2013 (*β* = −0.039, *p* < 0.01) and vice versa (*β* = −0.041, *p* < 0.01) at the between-person level. Additionally, there was no corresponding cross-lagged effect of cognitive function on hearing difficulty; rather, the more hearing difficulty, the greater the cognitive decline at the within-person level. According to the cross-lagged mediation model, depressive symptoms partially mediates the impact of cognitive function on subsequent hearing difficulty (indirect effect: −0.003, bootstrap 95% confidence interval: −0.005, −0.001, *p* < 0.05), but not the other way around.

**Conclusion:**

These results showed that within-person relationships between hearing impairment and cognitive function were unidirectional, while between-person relationships were reciprocal. Setting mental health first may be able to break the vicious cycle that relates hearing loss to cognitive decline. Comprehensive long-term care requires services that address depressive symptoms and cognitive decline to be integrated with the hearing management.

## 1 Introduction

One essential aspect of healthy aging is cognitive function (Oosterhuis Elise et al., 2022). The risk of cognitive decline increases rapidly with age, and middle-aged and older adults are more likely to experience it (Conte et al., 2022). By 2050, diseases related to cognitive decline are expected to affect over 152 million older people globally (Guerchet et al., 2020). The high incidence rate of cognitive decline has engendered substantial economic and caregiving encumbrances for families and society (Patterson, 2018). Research aims to identify modifiable risk factors for cognitive decline or dementia, as there are currently no effective treatment options (Livingston et al., 2020).

Hearing impairment is identified as the primary risk factor for dementia that can be modified in the population. It is responsible for 8% of all cases of dementia worldwide (Livingston et al., 2020). According to a recent systematic review, approximately 20% of the population may experience hearing loss, with individuals over the age of 50 accounting for 62.1% of all cases (Haile et al., 2021). Approximately 64.9% of older adults in China indicated having subpar or average auditory abilities (Heine et al., 2019). Notably, there is a frequent association between cognitive decline and hearing impairment in adults (Ma et al., 2022). The two prevalent geriatric symptoms are linked to future negative outcomes, including falls, disabilities, and even mortality, which impose a significant burden on public health (Amieva et al., 2018; Xiao et al., 2021; Liu et al., 2022; Zhang et al., 2023).

Prior research has consistently shown a connection between hearing impairment and cognitive decline (Liu et al., 2023; Moradi et al., 2023; Ying et al., 2023). However, the specific findings and the chronological relationship between the two are still uncertain. An umbrella study, comprising 11 systematic reviews, has revealed that hearing loss in older adults may potentially contribute to cognitive impairment or dementia (Ying et al., 2023). A systematic review, encompassing Chinese-speaking adults, revealed a regression coefficient of −0.48 for the correlation between self-reported hearing loss and cognition (Fu et al., 2023). The majority of the longitudinal studies incorporated in the analysis were sourced from the Chinese Longitudinal Health Longevity Survey. It is uncertain whether hearing loss occurs before a decline in cognitive function or if it is the other way around. Prior research on the correlation between hearing loss and cognitive decline has predominantly employed cross-sectional methodologies, yielding inconclusive findings (Rong et al., 2020; Sun et al., 2021; Zhao et al., 2021; Fuller-Thomson et al., 2022; Hoff et al., 2022; Tamblay et al., 2023). Therefore, the potential for reverse causality cannot be dismissed.

However, the majority of previous studies on this subject have only examined a particular one-way connection, resulting in inconsistent results. For example, a study conducted over a period of 3 years, involving 6,338 older adults, found no significant correlation between hearing impairment and a decline in cognitive function over time (Yorgason et al., 2022). Nevertheless, a study encompassing 5,721 adults aged 40 and above who exhibited normal cognitive abilities discovered that hearing loss amplified the likelihood of transitioning to mild cognitive impairment (Bucholc et al., 2022). The study employed a longitudinal design to investigate the impact of initial hearing loss on subsequent alterations in cognitive function. It is worth noting that only a limited number of studies have examined the interplay between hearing and cognitive function dynamics (Qiu et al., 2020; Fu et al., 2023; Matthews et al., 2023; Zhou et al., 2023; Zhou and Wu, 2023). Furthermore, according to the cognitive load hypothesis of perception, cognitive declines can result in diminished sensory performance as a result of a decrease in available sensory processing resources (Nixon et al., 2019). Hence, the orientation of this correlation may also be contradictory.

We found that there is a robust correlation between hearing impairment and increased susceptibility to cognitive decline in adults (Yang and Luo, 2024). Additionally, there is evidence suggesting that cognitive decline may impact hearing function (Valsechi et al., 2022), although the effect is typically minimal or lacks statistical significance (Armstrong et al., 2020). In recent times, scholars have underscored the importance of taking into account both inter-individual and intra-individual factors in order to gain a comprehensive understanding of vertical relationships (Hamaker et al., 2015; Orth et al., 2021). The between-person hypothesis posits that adults with more serious hearing difficulty are more prone to cognitive decline in comparison to their counterparts, and vice versa. The within-person hypothesis posits that when an individual experiences higher levels of hearing difficulty, they are more likely to experience cognitive decline. Similarly, when an individual is undergoing cognitive decline, their hearing function may deteriorate. The association between hearing impairment and cognitive function has been examined using the Cross-Lagged Panel Model (CLPM) (Armstrong et al., 2020). A notable limitation of the CLPM is its inability to differentiate between inter-individual effects (between-person effects) and intra-individual effects (within-person effects). It has been argued that the effects observed among individuals can only be generalized to the individual under very strict assumptions (Berry and Willoughby, 2017).

To examine associations between variables within individuals, researchers can employ the Random Intercept Cross-Lagged Panel Model (RI-CLPM), which is an extension of the CLPM (Hamaker et al., 2015). Thus far, Yu et al. have utilized the model to investigate the reciprocal association between hearing loss and loneliness at an individual level (Yu et al., 2021); however, no previous studies have examined the individual-level effects of hearing loss on cognitive function. So, it is important to do a large-scale, cross-lagged longitudinal study to see how much the results change when inter-individual effects and intra-individual effects are separated. If the results of an RI-CLPM were repeated in a CLPM with the same sample, it would show that the conclusions from earlier CLPM research are a good reflection of how things really work inside people. Nevertheless, if the findings are not able to be reproduced, it may be necessary to contemplate further conclusions regarding the specific relationship between hearing impairment and cognitive ability within an individual.

Studying the possible psychosocial mechanisms linking hearing difficulty and cognitive function can provide valuable insights for identifying and preventing risks. Prior research has documented a reciprocal association between hearing difficulties and depressive symptoms (Huang et al., 2022; Wu, 2022). A systematic review of 35 studies revealed that older adults have a higher likelihood of developing depressive symptoms linked to hearing loss (Lawrence et al., 2020). Given that the majority of current research consists of observational studies, it remains difficult to ascertain whether hearing loss directly causes depressive symptoms or if individuals experience depressive symptoms as a result of poor health, which in turn affects their perception of hearing negatively. Furthermore, depressive symptoms could potentially serve as a risk factor or early indication of cognitive decline (Muhammad and Meher, 2021). A decrease in cognitive function may be indicative of depressive symptoms (Chau et al., 2019). An observational study conducted in the UK involving 11,855 participants aged 50 years and older reveals a temporal association between cognitive function and depressive symptoms (Desai et al., 2020). Previous research suggests that depressive symptoms may play a significant role as a modifiable risk factor, linking hearing loss to potential cognitive decline in the future (Dawes et al., 2015; Rutherford et al., 2018; Cao et al., 2023). Nevertheless, hearing and cognitive function, along with depressive symptoms, exhibit dynamic fluctuations in adults, potentially resulting in a reverse causal relationship. The current study examines the research question by employing a cross-lagged mediation model to explore the involvement of depressive symptoms as a mediator in the reciprocal association between hearing difficulties and cognitive function in middle-aged and older individuals.

Hence, the main objective of this research was to examine the reciprocal relationship between hearing difficulty and cognitive function in middle-aged and older adults, both at the individual and group levels. This was achieved by employing a four-wave cross-lagged panel design and using a substantial sample that represents the entire nation. Furthermore, it assesses whether depressive symptoms acts as a mediator in a potential two-way relationship. Due to the absence of prior literature, mediation analysis is regarded as exploratory.

## 2 Methods

### 2.1 Sample

This study adheres to the Strengthening the Reporting of Observational Studies in Epidemiology (STROBE) reporting guideline for observational studies (von Elm et al., 2014). The data were obtained from the China Health and Retirement Longitudinal Study (CHARLS), which is a comprehensive survey conducted on a national scale to gather information about China’s middle-aged and older population (aged 45 and above). A random selection was made of 150 county-level administrative units from 28 provinces in China for the purpose of conducting a large-scale survey. The CHARLS included a total of 17,708 participants in 2011 during its first wave. Follow-up inquiries were carried out in 2013 (Wave 2), 2015 (Wave 3), and 2018 (Wave 4), respectively. This study examined the data obtained from CHARLS, spanning from Wave 1 in 2011 to Wave 4 in 2018. The CHARLS investigator inquired whether the respondents had received a medical diagnosis for memory-related diseases such as Alzheimer’s disease, brain atrophy, or Parkinson’s disease. If the respondents answered affirmatively, they were subsequently questioned about the timing of their diagnosis. In order to be included in the study, participants had to meet the following criteria: be 45 years of age or older, have provided four repeated measurements for hearing difficulty and cognition from Wave 1 to Wave 4, not have any memory-related diseases (such as Alzheimer’s disease, brain atrophy, or Parkinson’s disease) in Wave 1, and not have any missing data.

The Peking University Ethics Review Committee granted ethics approval for the CHARLS study (No. IRB00001052-11015). Every individual involved is required to complete and sign informed consent documents.

### 2.2 Cognitive function

The cognitive function test in CHARLS comprises two components: intelligence, with a score range of 0–10, and episodic memory, with a score range of 0–20.

The Telephone Interview of Cognitive Status (TICS) items and figure drawing were both used by the CHARLS to assess the intelligence state. The TICS assessed various components, such as the date (year, month, day), day of the week, performing serial subtraction of 7 from 100 (up to five times), and measuring orientation to time and attention (score range 0–9) (McArdle et al., 2007). Participants must create two overlapping five-star images of their exhibition using their drawing skills. The scoring scale for this task ranges from 0 to 1. Adding up the results of the aforementioned tests yields the intelligence state score.

The CHARLS used the CERAD (Consortium to Establish a Registry for Alzheimer’s Disease) version of immediate and delayed word recall to measure the episodic memory (Fillenbaum et al., 2008; Zhao et al., 2020). Episodic memory can be assessed through the recollection of familiar phrases, including immediate word recall (with a score range of 0–10) and delayed word recall (with a score range of 0–10), resulting in a total score of 20.

The cumulative cognitive function score is determined by summing the scores of all items (range: 0–30). A higher cognitive function score signifies superior cognitive function. The cognitive test module utilized in CHARLS has been confirmed by previous research (Burr et al., 2020; Hu et al., 2022; Xu et al., 2023).

### 2.3 Self-reported hearing status

The self-reported hearing was evaluated through a distinct questionnaire administered at the beginning and during the three subsequent visits. Participants were asked to rate their hearing as excellent, very good, good, fair, or poor, taking into account the use of a hearing aid if applicable. If the participant responds with ‘Excellent,’ they will receive 1 point. If the participant responds with ‘Very good,’ they will receive 2 points. If the participant responds with ‘Good,’ they will receive 3 points. If the participant responds with ‘Fair,’ they will receive 4 points. If the participant responds with ‘poor,’ they will receive 5 points. If the individual’s hearing was determined to be fair or poor, we inferred that they experienced hearing difficulty. This approach was employed in prior research utilizing CHARLS data (Xie et al., 2021; Ma et al., 2022).

### 2.4 Depressive symptoms

The evaluation of depressive symptoms was conducted using the 10-item Center for Epidemiologic Studies Depression (CES-D) scale. Participants were instructed to assess the frequency of their depressive symptoms over the past week using a scale ranging from 0 (indicating rare or no occurrence [< 1 day]) to 3 (indicating frequent or constant occurrence [5–7 days]). The overall score ranges from 0 to 30, with lower scores indicating a lesser degree of depressive symptoms.

### 2.5 Covariates

The covariates, such as age, sex, education attainment, marital status, health-related behavior, and chronic diseases, were collected during Wave 1 (Rehm et al., 2019; Zhang et al., 2022; Qian et al., 2023). Age was a continuous variable. Sex was classified into two categories: male and female. The level of education was categorized as either less than lower secondary, upper secondary & vocational training, or above. The marital status was classified into the following categories: married, partnered, separated, divorced, widowed, and never married. Health-related behaviors encompass smoking (current, former, or never) and alcohol consumption (current or never). Chronic diseases encompass conditions such as hypertension, diabetes, and cardiovascular diseases (Cichosz et al., 2020; Kelly and Rothwell, 2020).

### 2.6 Statistical analysis

Before conducting formal analysis, we utilized the independent sample *t*-test and the chi-square test to evaluate the differences in characteristics between the inclusion group and the exclusion group. We performed a normality test using a QQ plot, which is a graphical technique for evaluating the normality of a dataset. A QQ plot is a graphical tool that allows for the comparison of the sample distribution with the normal distribution. It achieves this by plotting the quantiles of the sample data against the corresponding quantiles of the normal distribution. The alignment of the points on the QQ plot with the diagonal line indicates that the data in our study exhibit a normal distribution. Hence, we employed frequency (%) to analyze categorical data and mean ± standard deviations (SDs) to analyze continuous data when examining the baseline characteristics.

Based on the hearing status data from Wave 4, we used independent sample *t*-tests and chi-square tests to analyze the variations in baseline characteristics. Respondents were classified into four groups according to the quartiles of their cognitive function scores at Wave 4. We employed independent sample *t*-tests to examine age disparities among cognitive function groups. We employed the Mann–Whitney test to assess disparities in cognitive function among individuals belonging to various gender, education level, marital status, alcohol consumption, hypertension, diabetes, and cardiovascular disease subgroups. We employed the Kruskal-Wallis H test to assess disparities in cognitive function among individuals with varying levels of smoking. Furthermore, Pearson’s correlation test was employed to examine the association between hearing difficulty, depressive symptoms, and cognitive function at the four-time points.

In order to investigate the two-way connections hearing difficulty and cognitive function, both at the individual level and within individuals, we utilized a cross-lagged panel model (CLPM) and a random-intercept cross-lagged panel model (RI-CLPM) for estimation.

First, we conducted the CLPM to investigate the reciprocal connections between hearing difficulty and cognitive function at the between-person level. This was achieved by constructing four distinct models. Model 1 was constructed without any modifications to estimate the overall impact. Model 2 was controlled for age, gender, educational attainment, and marital status. Model 3 underwent additional adjustments to account for smoking and drinking. Model 4 incorporates the covariates from Model 3, along with the inclusion of hypertension, diabetes, and cardiovascular diseases. We employed Models 2, 3, and 4 to examine whether covariates accounted for the impact of hearing difficulty on cognitive function (or the impact of cognitive function on hearing difficulty). The model system focused on the reciprocal influence between hearing impairment and cognitive ability. All variables observed in Waves 1 through 4 were incorporated into the model.

We computed stability paths, which represent the autoregression paths of the variables. Additionally, we calculated cross-lagged paths, which indicate the effect of one variable on another variable at the next wave. Finally, we determined the correlations between the variables. Furthermore, we established the control pathway for the initial wave covariates pertaining to the study variable. In the context of cross-lagged panel models (CLPM), the cross-lagged coefficient refers to the relationship between an individual’s higher or lower score on one variable compared to others and their subsequent change in rank order on another variable compared to others. Subgroup analyses were performed to account for the susceptibility of hearing difficulty and cognitive function to gender (male/female), education (low education level/high education), and age (45–59 years old/ ≥ 60 years old). Participants with an education level below lower secondary were categorized as the “low education” group, while those with an education level of upper secondary and vocational training, or higher, were categorized as the “high education” group.

To get a good estimate, we made the CLPM model better by testing all cross-lagged paths, stability paths, and correlations for time invariance. In our analysis, we compared the fit of different models. First, we had an unconstrained baseline model (Model 4). Then, we compared this model with Model 5, where all the cross-lagged paths were fixed to be time-invariant. Next, we compared Model 4 with Model 6, where all the stability paths were fixed to be time-invariant. We also compared Model 4 with Model 7, where all the correlated changes at Wave 2, Wave 3, and Wave 4 were fixed to be time-invariant. Finally, we looked at Model 4 and Model 8, where all the cross-lagged paths, stability paths, and Wave 2-Wave 4 correlated changes were set to not change over time. We also checked how different the model fits were by looking for three things: changes in *χ*2 (Δ*χ*2) that were statistically significant at a level of *p* < 0.05, changes in CFI (ΔCFI) with values lower than −0.010, and changes in RMSEA (ΔRMSEA) with values higher than 0.015 (Chen, 2007).

Furthermore, we performed the RI-CLPM analysis utilizing identical CLPM variables. This model is different from CLPM because it includes random intercepts that take into account how trait-level scores vary from person to person across all measurement points in the study variable (Hamaker et al., 2015). “Cross-lagged coefficient” in RI-CLPM means the chance that a short-term change in one variable’s deviation from the trait level will cause a change in another variable’s temporary deviation from the trait level (Orth et al., 2021). The mean scores of observed hearing difficulty and cognitive function were regressed on their respective latent factors, with each loading constrained to one. The observed variables’ residual variances were set to zero, enabling the latent factor structure to account for both the variance within individuals and the variance between individuals. Subsequently, we incorporated two arbitrary intercepts (one for hearing difficulty and the other for cognitive function) while constraining the factor loadings to a value of one. The random intercepts indicate the consistent and inherent variations among individuals in terms of their hearing difficulty and cognitive function. The correlation between the random intercepts indicated the extent to which consistent variations in hearing difficulty among individuals were associated with consistent variations in cognitive function (Hamaker et al., 2015).

The model fit indices used in our study were the comparative fit index (CFI) and root mean square error of approximation (RMSEA). A CFI value above 0.900 and an RMSEA value below 0.080 indicate a good fit.

**Figure 1:**
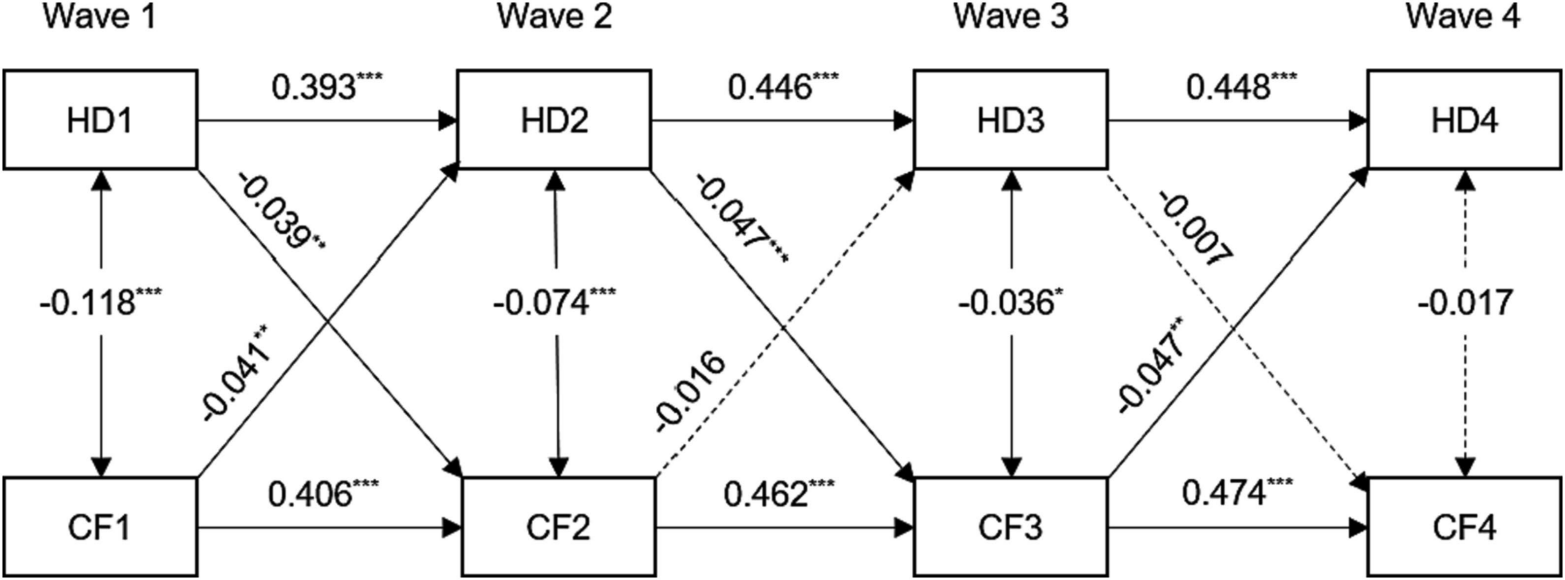
Standardized path diagram of cross-lagged panel model between hearing difficulty and cognitive function, CHARLS (*n* = 4,363), 2011–2018. For the sake of brevity, all covariates and residuals were estimated in the analysis but not shown in the diagram. Model adjusted for age, sex, education level, marital status, smoking, drinking, hypertension, diabetes, and cardiovascular diseases. HD1, HD2, HD3, and HD4 = hearing difficulty at Wave 1, 2, 3, 4; CF1, CF2, CF3, and CF4 = cognitive function at Wave 1, 2, 3, 4; ^***^p < 0.001; ^**^p < 0.01; ^*^p < 0.05.

The Cross-Lagged Mediation Model seeks to reveal the causal mechanisms that connect a predictor variable to an outcome variable (Preacher, 2015). This approach elucidates the precise channels by which the predictor variable impacts the outcome variable, offering a more profound comprehension of the fundamental causal mechanisms in action. Prior to calculating the longitudinal mediation model, three preconditions for mediation were determined using cross-lagged panel models based on Baron & Kenny’s causal steps approach (Baron and Kenny, 1986). (1) The presence of hearing difficulty is a longitudinal predictor of cognitive function, as shown in Figure 1. (2) The presence of hearing difficulty is a longitudinal predictor of depressive symptoms, as shown in Supplementary Figure S4. (3) depressive symptoms is a longitudinal predictor of cognitive function, as shown in Supplementary Figure S4. During each stage, we also assessed the existence of reverse pathways to ascertain their potential influence.

In this study, a cross-lagged mediation model was constructed to determine the indirect influence of depressive symptoms on the longitudinal association between hearing impairment and cognitive performance. The bootstrap method with bias correction (5,000 draws) evaluated the significance of indirect effects (Mackinnon et al., 2004). If the 95% bootstrap confidence interval (CI) included zero, then the indirect effect was deemed insignificant. Standardized path coefficients and 95% CI were reported to compare the magnitude of the predicted effects and determine which variable has a more significant effect on the other. The mediation ratio is calculated by dividing the indirect effect by the total effect.

The analyses were performed in SPSS version 26.0, with the exception of cross-lagged path analysis, which was carried out using Mplus 7.4. For all analyses, *p* < 0.05 was statistically significant.

## 3 Results

The screening procedure of the study participants is depicted in Supplementary Figure S1. The Chinese Health and Retirement Longitudinal Study, conducted in 2011, initially enrolled a total of 17,708 participants. After applying the specified criteria, a total of 5,471 participants were deemed ineligible and therefore excluded. During Wave 2, a total of 1,494 participants were no longer available for further observation or data collection. In addition, the study excluded 2,018 individuals who were not evaluated for hearing and cognitive abilities. During Wave 3, a total of 760 individuals were unable to be tracked, and an additional 770 individuals were excluded from the study because they were not evaluated for their hearing and cognitive abilities. Wave 4 saw a loss of 682 individuals who could not be tracked, and 2,150 individuals were excluded from the study due to not meeting the criteria for inclusion. The study included a total of 4,363 participants after applying all the selection criteria.

Supplementary Figure S1 indicates that a grand total of 13,345 individuals were excluded. In comparison to the sample that was included, the sample that was excluded exhibited the following characteristics: it was older, had a greater percentage of females, a higher percentage of individuals with low educational attainment, a lower percentage of married individuals, a lower percentage of individuals who consumed alcohol, a lower percentage of individuals who smoked cigarettes, and a higher percentage of individuals with high blood pressure (all *p* < 0.05, Supplementary Table S1). There was no significant disparity in the prevalence of diabetes and cardiovascular diseases.

Out of the 4,363 participants included in the analysis, 44.83% were females. The mean (SD) age was 56.16 (7.75) years. Supplementary Table S2 illustrates that individuals who reported hearing difficulties at the follow-up (Wave 4) were characterized by older age, lower educational attainment, a higher prevalence of cardiovascular disease, and exhibited inferior hearing status and cognitive function at baseline as compared to those without hearing difficulties. According to statistical analysis, there was a noticeable and significant difference in the participants’ ages in different subgroups based on cognitive function. Based on the non-parametric test, individuals who identified as female, had a high level of education, were married, and were non-smokers at the beginning of the study showed significantly higher levels of cognitive function in Wave 4. These results were statistically significant with a *p*-value of less than 0.05, as shown in Supplementary Table S2.

The bivariate correlations among hearing difficulty, depressive symptoms, and cognitive function are documented in Supplementary Table S3. The study found a positive correlation between hearing difficulty and depressive symptoms, with correlation coefficients ranging from 0.134 to 0.222. Additionally, there was an inverse correlation between hearing difficulty and cognitive function, with correlation coefficients ranging from −0.085 to −0.139. These correlations were observed consistently at each wave and across multiple waves. A more severe hearing difficulty at Wave 1 was linked to increased depressive symptoms levels at Wave 1 and decreased cognitive function at Wave 2. There was a correlation between a higher depressive symptoms score and lower cognitive function at each time point (all *p* < 0.001; see Supplementary Table S3).

### 3.1 Between-person associations

We investigated the correlations between hearing impairment and cognitive performance using the CLPM. The adjustment for various covariates at the beginning of the study reduced the impact of the estimated effects but did not change most of the conclusions. However, the significant relationship between cognitive function in Wave 2 and hearing difficulty in Wave 3, as well as the significant relationship between hearing difficulty in Wave 3 and cognitive function in Wave 4, disappeared after the adjustment. In addition, the relationships between the variables and the accuracy of the model remained consistent even when different factors were taken into account (see Table 1).

**Table 1:** Model fit indices and standardized path coefficients for cross-lagged models between hearing difficulty and cognitive function, CHARLS (*n* = 4,363), 2011–2018.

Paths	Model 1		Model 2		Model 3		Model 4	
	*β*	SE	*β*	SE	*β*	SE	*β*	SE
Autoregressive paths								
HD1 → HD2	0.403^***^	0.013	0.395^***^	0.013	0.395^***^	0.013	0.393^***^	0.013
HD2 → HD3	0.455^***^	0.012	0.448^***^	0.012	0.447^***^	0.012	0.446^***^	0.012
HD3 → HD4	0.498^***^	0.011	0.490^***^	0.012	0.489^***^	0.012	0.448^***^	0.015
CF1 → CF2	0.448^***^	0.012	0.406^***^	0.013	0.406^***^	0.013	0.406^***^	0.013
CF2 → CF3	0.510^***^	0.011	0.462^***^	0.012	0.462^***^	0.012	0.462^***^	0.012
CF3 → CF4	0.538^***^	0.011	0.476^***^	0.012	0.475^***^	0.012	0.474^***^	0.012
Cross-lagged paths								
HD1 → CF2	−0.053^***^	0.014	−0.038^**^	0.013	−0.038^**^	0.013	−0.039^**^	0.013
CF1 → HD2	−0.057^***^	0.014	−0.040^**^	0.014	−0.041^**^	0.014	−0.041^**^	0.014
HD2 → CF3	−0.064^***^	0.013	−0.046^***^	0.013	−0.046^***^	0.013	−0.047^***^	0.013
CF2 → HD3	−0.041^**^	0.013	−0.016	0.014	−0.016	0.014	−0.016	0.014
HD3 → CF4	−0.027^*^	0.013	−0.005	0.013	−0.006	0.013	−0.007	0.013
CF3 → HD4	−0.066^***^	0.013	−0.045^**^	0.014	−0.045^**^	0.014	−0.047^**^	0.014
Residual correlations								
HD1 with CF1	−0.139^***^	0.015	−0.117^***^	0.015	−0.117^***^	0.015	−0.118^***^	0.015
HD2 with CF2	−0.086^***^	0.015	−0.073^***^	0.015	−0.073^***^	0.015	−0.074^***^	0.015
HD3 with CF3	−0.051^**^	0.015	−0.035^*^	0.015	−0.035^*^	0.015	−0.036^*^	0.015
HD4 with CF4	−0.034^*^	0.015	−0.014	0.015	−0.015	0.015	−0.017	0.015
Model fit indices								
CFI	0.817		0.848		0.848		0.849	
TLI	0.589		0.238		−0.063		−0.356	
SRMR	0.089		0.058		0.047		0.040	
RMSEA	0.175		0.168		0.168		0.168	

Model 1 was built without any adjustments; Model 2 is adjusted for age, sex, education level, marital status; Model 3 is adjusted for the covariates in model 2 plus smoking, drinking; Model 4 is adjusted for the covariates in model 3 plus hypertension, diabetes, and cardiovascular diseases. ^***^p < 0.001; ^**^p < 0.01; ^*^p < 0.05. HD1, HD2, HD3, and HD4 = hearing difficulty at Wave 1, 2, 3, 4; CF1, CF2, CF3, and CF4 = cognitive function at Wave 1, 2, 3, 4.

The standardized path coefficients for the final model (Model 4) are illustrated in Figure 1. The fit indices of this model were unsatisfactory, with a Comparative Fit Index (CFI) of 0.849, a Standardized Root Mean Square Residual (SRMR) of 0.040, and a Root Mean Square Error of Approximation (RMSEA) of 0.168. The associations between hearing difficulty and cognitive function at each time point were statistically significant and demonstrated inverse relationships (except for Wave 4). The subsequent autoregressive effects of hearing difficulty and cognitive function exhibited statistically significant differences (all *p* < 0.001). The standardized stability path estimates for hearing difficulty (range from 0.393 to 0.448) and cognitive function (range from 0.406 to 0.474) were consistently stable.

The cross-lagged effects analysis revealed a significant association between hearing difficulty at Wave 1 and a lower cognitive function score at Wave 2 (*β* = −0.039, *p* < 0.01). Similarly, a significant association was found between a lower cognitive function score at Wave 1 and hearing difficulty at Wave 2 (*β* = −0.041, *p* < 0.01). Similarly, the presence of hearing impairment during Wave 2 was found to be a significant predictor of impaired cognitive function during Wave 3 (*β* = −0.047, *p* < 0.001). Nevertheless, there was no significant correlation between hearing impairment at Wave 3 and cognitive performance at Wave 4.

In Supplementary Table S4, when all cross-lagged paths or all Wave 2–Wave 4 correlated changes are fixed to time-invariant, Model 5 and Model 7 did not make the model fit significantly worse than Model 4, which was not constrained in any way. This indicates no developmental difference in the relationship between hearing difficulties and cognitive function. When all stability paths are fixed to be time-invariant, Model 6 made the model fit significantly worse than an unconstrained model (Model 4). This indicates that hearing difficulties and cognitive function are time-varying and can affect their interaction relationship. Model 8, which was the most parsimonious, resulted in a significantly worse fit compared to an unconstrained model. None of the eight models for the CLPM were very well fitted, so we did not fix the cross-lagged paths, stability paths, and all Wave 2–Wave 4 correlated changes to be time-invariant.

We further compared differences in standardized path coefficients to examine the magnitude of the cross-lagged relationship between hearing difficulty and cognitive function from Wave 1 to Wave 2. The study revealed that the impact of cognitive function on future hearing difficulty was significantly greater than the impact of hearing difficulty on future cognitive function (Wald *χ*2 (1) = 7.241, *p* < 0.01).

**Figure 2:**
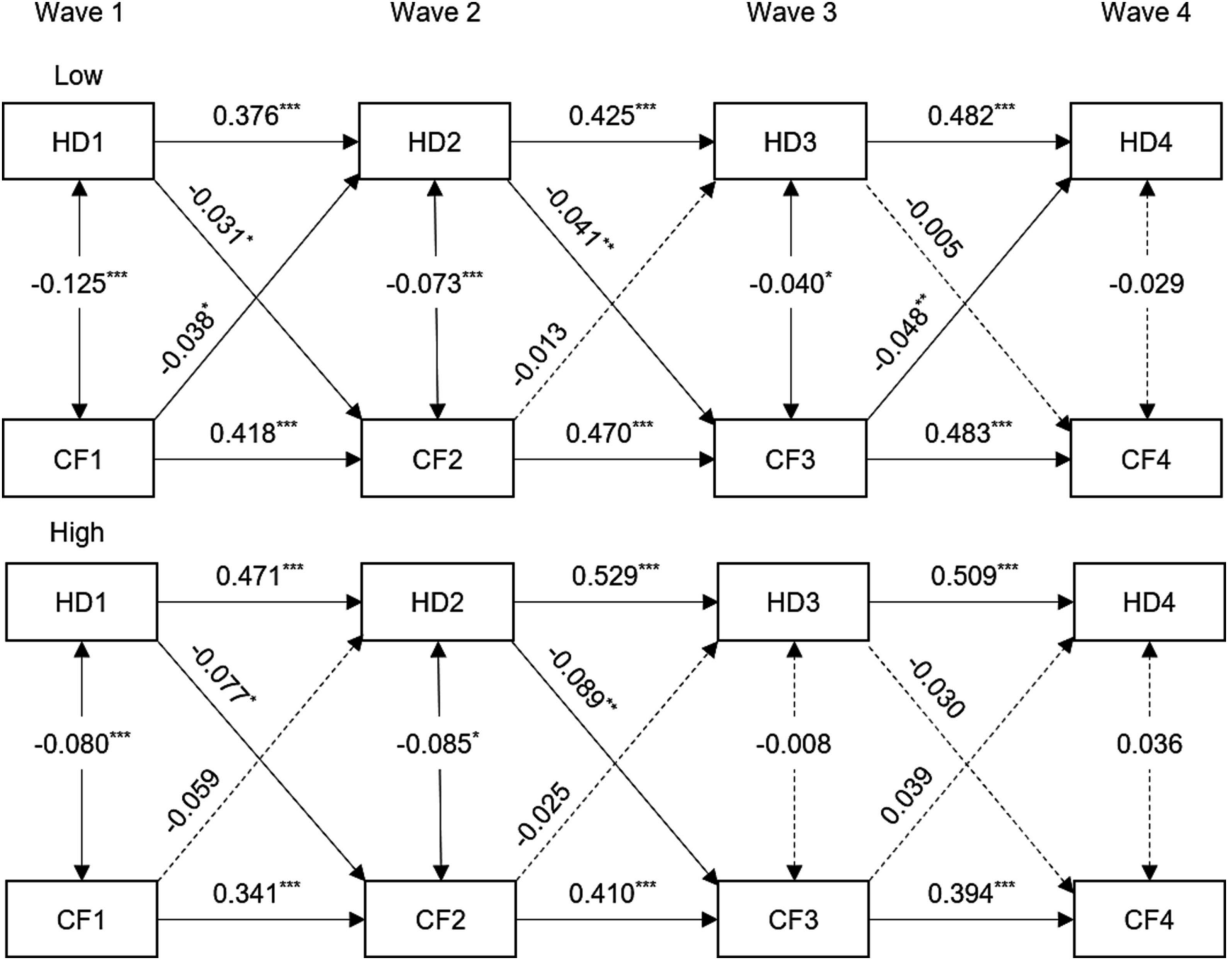
Standardized coefficient estimates for the bidirectional longitudinal association between hearing difficulty and cognitive function by stratified by education, CHARLS, 2011–2018. For simplicity, all covariates and residuals were estimated in the analysis but not shown in the diagram. Model adjusted for age, gender, marital status, smoking, drinking, hypertension, diabetes, and cardiovascular diseases. HD1, HD2, HD3, and HD4 = hearing difficulty at Wave 1, 2, 3, 4; CF1, CF2, CF3, and CF4 = cognitive function at Wave 1, 2, 3, 4. ^***^p < 0.001; ^**^p < 0.01; ^*^p < 0.05.

When analyzing the data based on gender, we found that the relationship between hearing difficulty and cognitive function remained in females from Wave 1 to Wave 2. However, we only observed a significant one-way association in males. You can refer to Supplementary Figure S2 and Supplementary Table S5 for more details. Within the low-education population, the subgroup analysis revealed a persistent bidirectional association between hearing difficulty and cognitive function. However, there was only a clear one-way connection observed in the population with a high level of education from Wave 1 to Wave 3 (see Figure 2 and Supplementary Table S6). The subgroup analysis revealed a clear and consistent bidirectional relationship between hearing difficulty and cognitive function within the 45–59 age group, as shown in Supplementary Figure S3 and Supplementary Table S7. However, the correlation between the two is diminished or completely disappeared in the older population.

### 3.2 Within-person associations

We investigated the correlations between hearing difficulty and cognitive function using the RI-CLPM. The models that incorporate autoregressive and lagged path coefficients invariant constraints have good model fits. This article reports the results of RI-CLPM with autoregressive and lagged path invariant constraints. As shown in Supplementary Table S8, the most parsimonious model (Model 8) made the model fit significantly worse than an unconstrained model (Model 4). Model 5 did not make the model fit significantly worse than an unconstrained model (Model 4). Consequently, we did not constrain stability paths, and Wave 2 - Wave 4 correlated changes to be time-invariant, excluding the cross-lagged paths.

**Figure 3:**
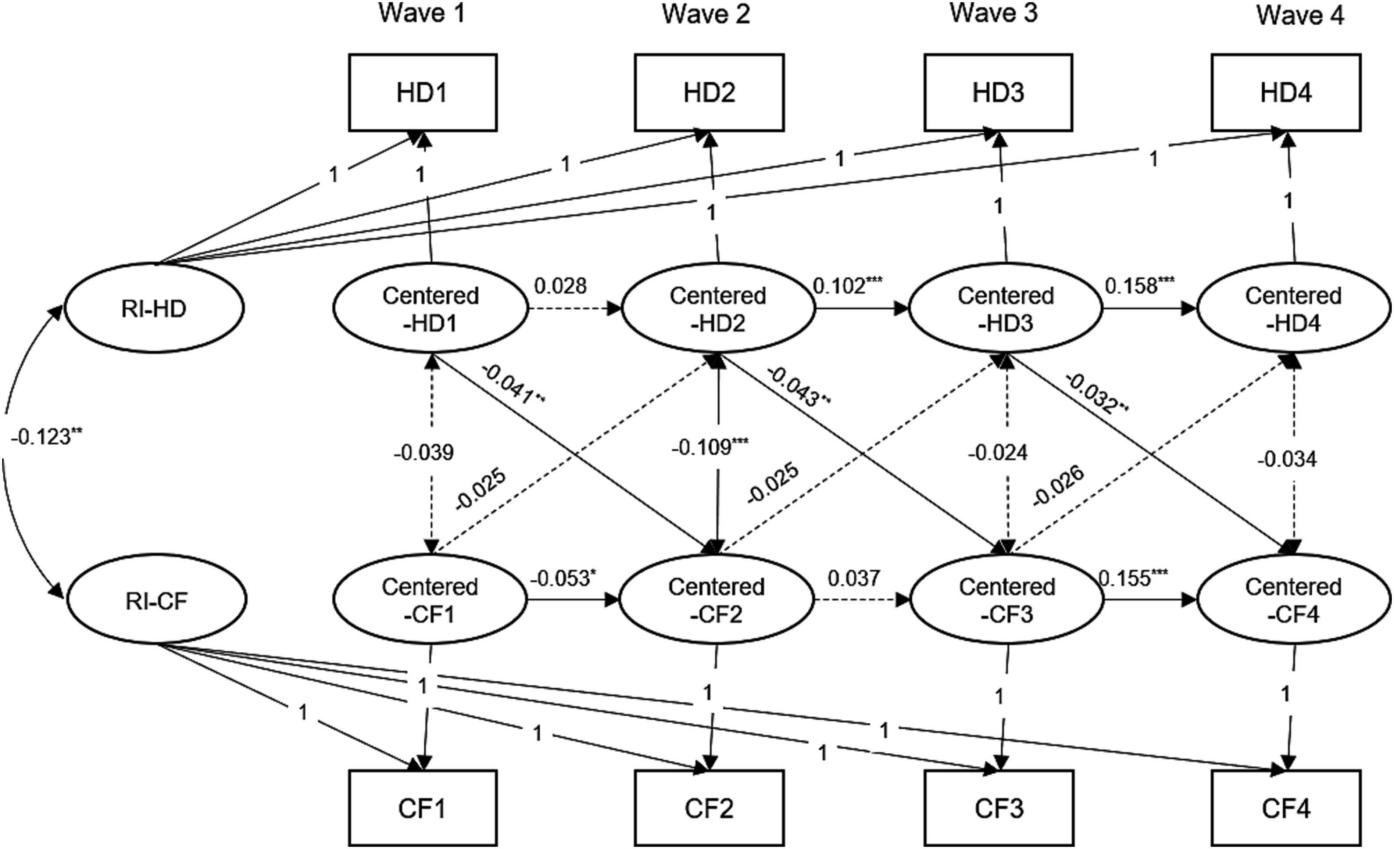
Random-intercept cross-lagged panel model results for the hearing difficulty and cognitive function, CHARLS, 2011–2018. For the simplicity, all covariates and residuals were estimated in the analysis but not shown in the diagram. Model adjusted for age, gender, marital status, smoking, drinking, hypertension, diabetes, and cardiovascular diseases. Model with all cross-lagged paths fixed to be time-invariant. HD1, HD2, HD3, and HD4 = hearing difficulty at Wave 1, 2, 3, 4; CF1, CF2, CF3, and CF4 = cognitive function at Wave 1, 2, 3, 4. RI = random intercepts; ^***^p < 0.001; ^**^p < 0.01; ^*^p < 0.05.

The cross-lagged and stability path coefficients are shown in Supplementary Table S9. As shown in Table 1 and Supplementary Table S9, the fits of the RI-CLPM were better than the CLPM, as indicated by CFI and RMSEA. Regarding the longitudinal associations linking hearing difficulty and cognition at the within-person level, we found that there were no significant effects of cognition on hearing difficulty. However, we did find that hearing difficulty can predict cognitive decline in the next stage (range from −0.032 to −0.043, *p* < 0.05). Figure 3 shows the standardized model results for hearing difficulty and cognition. We also explored the initial associations between the random intercepts of the study variables. As shown in Figure 3, the initial correlation between hearing difficulty and cognition was not significant. Furthermore, there was a significant correlation between the initial level of self-rated hearing difficulty and the initial level of cognition (*β* = −0.123, SE = 0.028, *p* < 0.001). This indicates that individuals with hearing difficulty experienced cognitive decline at a higher frequency at baseline. In summary, our research revealed that hearing difficulty has a negative impact on cognitive improvement at an individual level rather than showing a two-way relationship.

### 3.3 Mediation analysis

In order to examine the internal mechanism of the relationship between hearing difficulty and cognitive function, we conducted additional research to confirm the role of depressive symptoms as a mediator.

Before conducting the formal mediation analyses, we examined the reciprocal associations between hearing difficulty and depressive symptoms, depressive symptoms and cognitive function, and hearing difficulty and cognitive function. These analyses were necessary prerequisites for the longitudinal mediation analyses. Supplementary Figure S4 demonstrates a significant correlation between hearing difficulty and depressive symptoms, with both conditions predicting each other at the specified time points (all *p* < 0.05). Statistically significant predictions were found for both depressive symptoms and cognitive function at the specified time points (all *p* < 0.05). As previously examined, there could be a reciprocal association between hearing impairment and cognitive ability.

**Figure 4:**
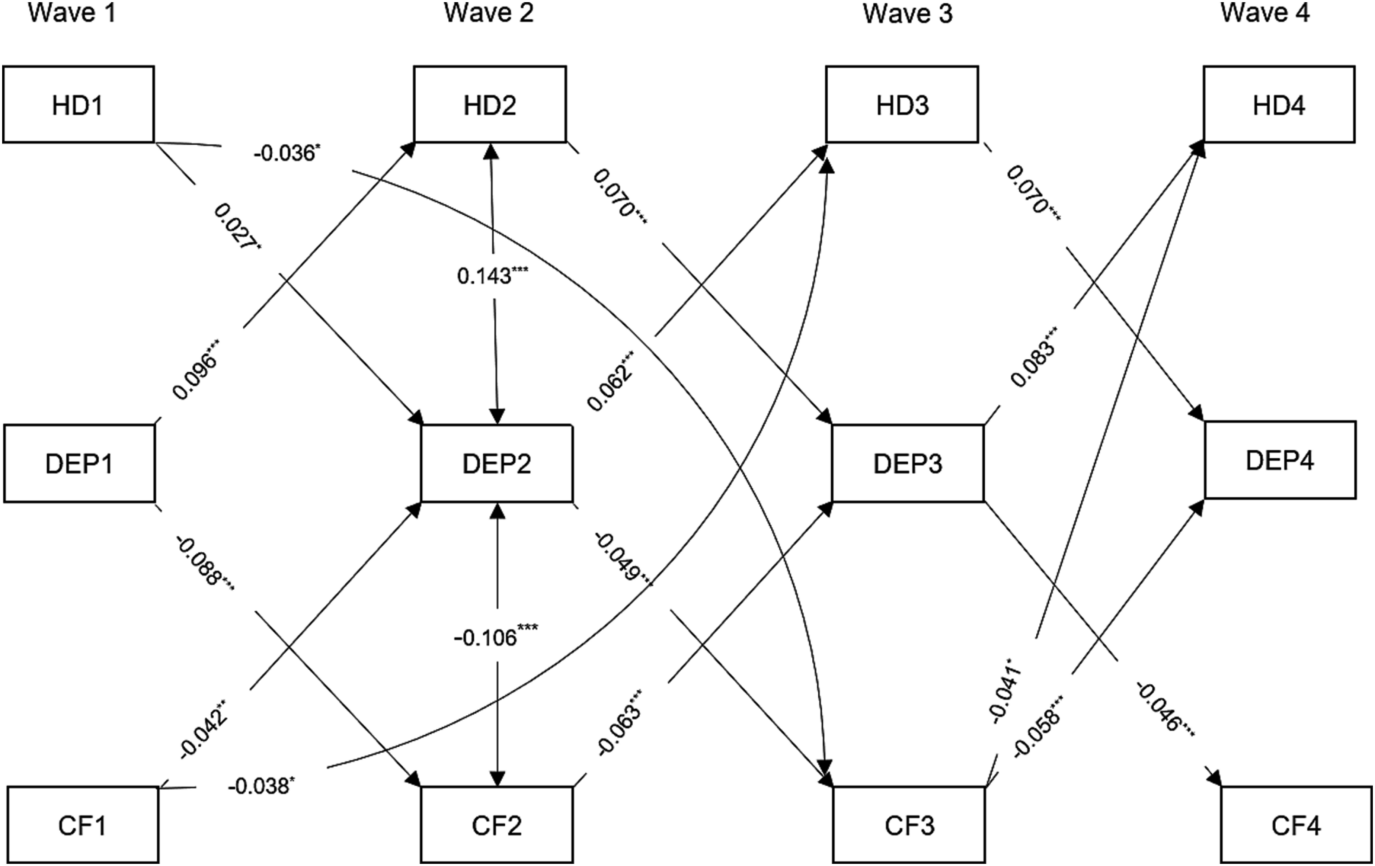
Standardized path diagram of cross-lagged mediation model, CHARLS, 2011–2018. Covariates including age, gender, marital status, smoking, drinking, hypertension, diabetes, and cardiovascular diseases were estimated in the analysis but not shown in the diagram. Only statistically significant cross-lagged path coefficients are shown in the diagram. HD1, HD2, HD3, and HD4 = hearing difficulty at Waves 1, 2, 3, 4; CF1, CF2, CF3, DEP1, DEP2, DEP3, and DEP4 = depressive symptoms at Wave 1, 2, 3, 4, and CF4 = cognitive function at Wave 1, 2, 3, 4. ^***^p < 0.001; ^**^p < 0.01; ^*^p < 0.05.

The standardized path coefficients for the cross-lagged mediation model are presented in Figure 4 and Supplementary Table S10. The findings indicated that depressive symptoms at Wave 2 mediated 7.32% of the relationship between cognition at Wave 1 and self-report hearing difficulty at Wave 3 The indirect effect was estimated at *β* = −0.003, with a bootstrap 95% confidence interval of −0.005 to −0.001. The direct effect was estimated at *β* = −0.038, with a bootstrap 95% confidence interval of −0.062 to −0.013. However, the presence of hearing difficulties at Wave 2 mainly affect cognitive function at Wave 4 through depressive symptoms at Wave 3, and vice versa. Conversely, the indirect effect of self-report hearing difficulty at Wave 1 on cognition at Wave 3 through depressive symptoms at Wave 2 was not significant (Indirect effect: *β* = −0.001, bootstrap 95% CI: −0.003, 0.000).

## 4 Discussion

Our study is the first to establish a two-way connection between hearing difficulty and cognitive function. The findings are based on a comprehensive longitudinal study of a representative sample of Chinese adults aged 45 and above. First, we discovered a detrimental correlation between hearing difficulty and cognitive function on a reciprocal basis, specifically at the between-person level. Although there was a correlation between hearing difficulty and cognitive decline at the within-person level, cognitive function did not serve as a predictor of hearing difficulty. Second, we noticed that the influence of cognitive function on hearing difficulty is more significant than the influence of hearing difficulty on cognitive function. This suggests that cognitive function plays a dominant role in the two-way relationship between hearing difficulty and cognitive function at the between-person level. Third, we discovered that depressive symptoms partially mediated the cognition-to-hearing difficulty association. The hearing impairment observed in middle-aged and older adults with low cognitive function can be attributed, at least in part, to the deterioration of depressive symptoms, a previously unidentified factor.

The findings from the cross-lagged panel model indicate a potential reciprocal association between hearing impairments and cognitive abilities, which diminishes as individuals grow older. Supplementary Figure S3 demonstrates a reciprocal association between hearing impairments and cognitive abilities in individuals aged 45–59 years, while this relationship was not observed in older individuals. Hearing loss and cognitive function exhibit a parallel progression as one ages (Tran et al., 2021), potentially concealing the interdependent influences of both. The bidirectional association demonstrated some degree of consistency with prior research, although these associations have only been validated independently and in a unidirectionally manner. The majority of these studies provided evidence that hearing difficulty predicts cognitive decline (Amieva et al., 2015; de la Fuente et al., 2019; Armstrong et al., 2020; Slade et al., 2020; Barbosa et al., 2023). A systematic review of 35 studies revealed a strong correlation between hearing loss and an increased propensity for depression in older individuals (Lawrence et al., 2020). The sensory deprivation hypothesis posits that age-related hearing loss, leading to prolonged sensory deprivation, has a persistent detrimental impact on both the structure and function of the brain (Powell et al., 2021). Wang et al.’s neuroimaging analysis revealed a strong link between impaired hearing and decreased volume in specific areas of the temporal cortex, such as the superior temporal auditory association cortical areas, hippocampus, and precuneus (Wang et al., 2022).

Furthermore, older individuals may experience difficulties hearing amidst background noise due to impaired executive function and fluid memory capabilities. This can lead to increased susceptibility to distractions from new auditory or visual stimuli, resulting in a reduced ability to concentrate on words spoken by a single speaker. Consequently, individuals may mistakenly perceive themselves as having a hearing impairment (Anderson et al., 2013). A systematic review examined the frequency of hearing loss in individuals with mild cognitive impairment. The analysis included data from three cross-sectional studies, which revealed relative risk values as high as 1.44; however the sample size of included studies was small (Lau et al., 2022). A UK study conducted over a period of 12 years, involving individuals over the age of 50, found that changes in recall memory were indicative of hearing loss. The study revealed that individuals in the group with the lowest recall memory trajectory were seven times more likely to develop hearing impairment compared to those in the group with the highest recall memory trajectory. It is important to note that the study had a lengthy follow-up period, did not assess hearing in stages, and could not establish a causal relationship between cognitive trajectory and hearing impairment at the final follow-up (Maharani et al., 2020). Nevertheless, a prior investigation conducted by the Baltimore Longitudinal Study of Aging (BLSA) scrutinized this correlation using a traditional CLPM and concluded that cognitive performance was not indicative of changes in hearing ability (Armstrong et al., 2020). Possible factors contributing to this could be the limited duration of the follow-up, the relatively small size of the sample, and the participation of older individuals in the study (mean age at the start: 73.9 ± 8.0). Unlike previous studies, ours had a large sample size and four follow-up visits, which provided strong evidence to support the investigation of the two-way relationship between hearing impairments and cognitive performance. Consequently, it is crucial to conduct screening and evaluation of hearing and cognitive abilities in middle-aged and elderly individuals to ensure their overall well-being. Further investigation is required to expand knowledge in this field.

As far as we know, our study is the first to utilize the RI-CLPM to investigate the causal connection between hearing impairments and cognitive ability. The results of the RI-CLPM, which did not show any impact of cognitive function on hearing difficulty, moderate the conclusions drawn from the CLPM. It has been found that the cross-lagged relationships found in a cross-lagged panel model (CLPM) are not as strong when they are repeated in a random-intercept cross-lagged panel model (RI-CLPM) (Etherson et al., 2022). The within-person process demonstrates that changes in cognitive function do not predict changes in the degree of hearing difficulty. The common-cause hypothesis says that both hearing and cognitive impairment can be caused by the same underlying factors, such as inflammation, vascular pathology, and other systemic neurodegenerative processes that hurt the central nervous system. This can lead to a lack of correlation between the two conditions (Uchida et al., 2019). The common-cause hypothesis posits that there is a simultaneous decline in multiple sensory patterns and cognition. One account alone is insufficient to account for all the data, suggesting the presence of multiple mechanisms (Jafari et al., 2021). Thus, cognitive decline does not necessarily lead to increased hearing difficulty. It is important to differentiate between analyzing differences between individuals and within individuals. However, to fully understand the continuous development of transactional effects over time, it is necessary to enhance individual assessments and expand the range of assessment timing (Voelkle and Oud, 2013). Furthermore, further investigation is needed to comprehend the correlation between hearing and cognitive function in middle-aged and older adults requires more research. Ultimately, these future studies have the potential to advance the personalization of interventions in practical applications.

Another notable discovery from the current study is that the impact of prior cognitive function on subsequent hearing difficulty is more significant than that of prior hearing difficulty on subsequent cognitive function at the between-person level. The results of our study are backed by a longitudinal investigation involving 8,895 individuals aged 50 years and older. The purpose of the study was to determine if cognitive impairment occurs before self-reported hearing difficulties. The study found that individuals who experienced cognitive impairment were more likely to report poor hearing later on Valsechi et al. (2022). A plausible reason for this is that changes in cognitive function have a more immediate and visible impact on self-perceived hearing levels. On the other hand, it takes a while for the negative effects of hearing difficulty to become apparent in terms of cognitive function. A meta-analysis study has demonstrated that the rate of progression of subjective cognitive decline into mild cognitive impairment or dementia increases with age at baseline (An et al., 2024). The sample size included in this study was young and the rate of progression of cognitive decline may be low. The utilization of a sole question to assess hearing difficulty in this study resulted in restricted value ranges, creating a ceiling effect that limited the variability of reported hearing difficulties. The use of a single question to evaluate hearing difficulty in this study led to limited value ranges, causing a ceiling effect that restricted the range of reported hearing difficulties. A solitary entry might underestimate the impact of hearing impairments on cognitive function. These findings suggest that taking proactive measures to prevent and detect cognitive decline at an early stage may have a stronger protective effect on the overall health of individuals experiencing hearing difficulty.

Furthermore, the present study has identified the mediating mechanism for this association. Adults with cognitive decline can increase their self-assessment hearing status by reducing depressive symptoms. Notably, previous studies have confirmed the mediating pathways identified in our study. An example of this is a recent study conducted in the United Kingdom, which involved 11,855 participants aged 50 years and older. The study found that there was a two-way relationship between depressive symptoms and certain aspects of cognitive function over a 12-month period (Desai et al., 2020). These findings align with our own results for the first part of the mediation pathway, which focuses on the impact of cognitive function on depressive symptoms. As predicted, we discovered a reliable two-way connection between cognitive function and depressive symptoms from Wave 1 to Wave 4. One explanation for the connection between cognitive decline and depressive symptoms is that psychological pain is a response to subjective experiences of cognitive decline. For example, some studies have shown that cognitive decline can either hasten the onset of depressive symptoms or be present alongside depressive symptoms (Ganguli et al., 2006).

Moreover, there is a growing body of evidence supporting the connection between depressed mood and hearing impairment, which reinforces the second part of our mediation pathway (from depressive symptoms to hearing difficulty) (Kim et al., 2020; Wu, 2022). The two-way link between hearing loss and depressive symptoms has been looked at using logistic regression and two sets of longitudinal analyses from the CHARLS database (Wave 1 and Wave 3) (Wu, 2022). We enhance this perspective by employing a cross-lagged modeling analysis in our study. Patients with depressive symptoms may face an elevated risk of sudden sensorineural hearing loss due to heightened inflammation in the nervous system, impaired auditory processing, and associated comorbidities (Kim et al., 2020). In conclusion, alleviating depressive symptoms in middle-aged and older adults could potentially ameliorate subjective perception of hearing abilities in individuals experiencing cognitive decline.

This study exhibits several strengths. This study utilized the CLPM and RI-CLPM to examine the chronological connection between hearing difficulties and cognitive function in a sample of middle-aged and older individuals in China, representative of the entire nation. Furthermore, cognitive function was identified as the preceding factor in a two-way connection. Implementing early screening and preventive measures for cognitive function can have positive effects on the health of middle-aged and older adults. Furthermore, the presence of longitudinal repeated measurement data is an essential requirement for elucidating the causal relationships in mediating analysis. Through the implementation of a longitudinal mediation design, we have discovered that depressive symptoms partially serves as a mediator for the negative impact of cognitive function on hearing difficulties.

However, it is important to take into account certain restrictions. Initially, the assessment of hearing difficulty, depressive symptoms, and cognition relied on self-reported data, which may potentially introduce recalling bias. To minimize recall bias, the CHARLS project team conducted surveys with all participants using a consistent and standardized questionnaire. Additionally, the data collectors were kept unaware of the research hypotheses of the study. While a single item is typically used to assess hearing difficulties, research suggests that self-reporting may be the most effective method for evaluating functioning (Pope and Sowers, 2000). This is because objective measurements, such as pure tone threshold audiometry tests, fail to include how individuals make adjustments for their hearing impairments. While the measurement methods for these variables have been previously validated, measurement errors and deviations from commonly used methods may still occur. The cognitive screening tool in the population-based longitudinal study may only capture limited variability in the normal aging population. This, along with its “ceiling effect,” could lead to a lower estimate of the link between hearing loss that comes with getting older and cognitive decline. Future research may undertake a comparison of the impacts of diverse methods of evaluating auditory and cognitive abilities (relying on subjective reports versus utilizing objective measures) on their associations in individuals of different age groups. Furthermore, our study focused solely on one potential mechanism and discovered that while there were statistically significant mediating effects of depressive symptoms, they only accounted for a minor portion of the overall influence of cognition on hearing difficulty. This finding suggests the presence of additional significant factors that have not been thoroughly investigated in the sequence of events connecting cognitive processes to auditory perception. Additional investigation is required to examine these mediators. Furthermore, the cross-lagged model encompasses the inclusion of all individuals measured at four distinct time points, leading to a notable decrease in the sample size. Consequently, there is a possibility that selection bias could impact the research findings. Future research should be cautious and avoid increasing attrition rates in longitudinal surveys.

## 5 Conclusion

Our research reveals a reciprocal and detrimental association between hearing difficulties and cognitive function in middle-aged and older Chinese adults at the between-person level. In addition, cognitive function exerts a prominent influence in this bidirectional relationship. Depressive symptoms has an impact on the potential relationship between hearing difficulties and cognitive function. Preserving mental health performance can potentially disrupt the harmful cycle linking cognitive decline and hearing impairments. Intervention research aimed at preventing hearing loss should take into account elements that can postpone cognitive decline. Simultaneously, interventions aimed at preserving cognitive function may involve addressing the susceptibility to hearing impairments in individuals who are middle-aged and older.

## Data availability statement

The datasets presented in this study can be found in online repositories. The names of the repository/repositories and accession number(s) can be found at: http://charls.pku.edu.cn/.

## Ethics statement

The studies involving humans were approved by Peking University Ethics Review Committee. The studies were conducted in accordance with the local legislation and institutional requirements. The participants provided their written informed consent to participate in this study.

## Author contributions

XL: Writing – original draft, Conceptualization, Formal analysis, Investigation, Methodology, Project administration. MH: Conceptualization, Writing – review & editing. YZ: Writing – review & editing. RP: Writing – review & editing. YG: Writing – review & editing. CZ: Writing – review & editing. JH: Writing – review & editing. HF: Conceptualization, Writing – review & editing. MS: Writing – review & editing.
