# Supplementary material for: Bidirectional associations between hearing difficulty and cognitive function in Chinese adults: a longitudinal study
Source: Front Aging Neurosci. 2023 Dec 13;15:1306154. doi: 10.3389/fnagi.2023.1306154 (PMC10751337; doi:10.3389/fnagi.2023.1306154)
Supplement: Supplementary file 1 [file Data_Sheet_1.docx]

**Supplement**


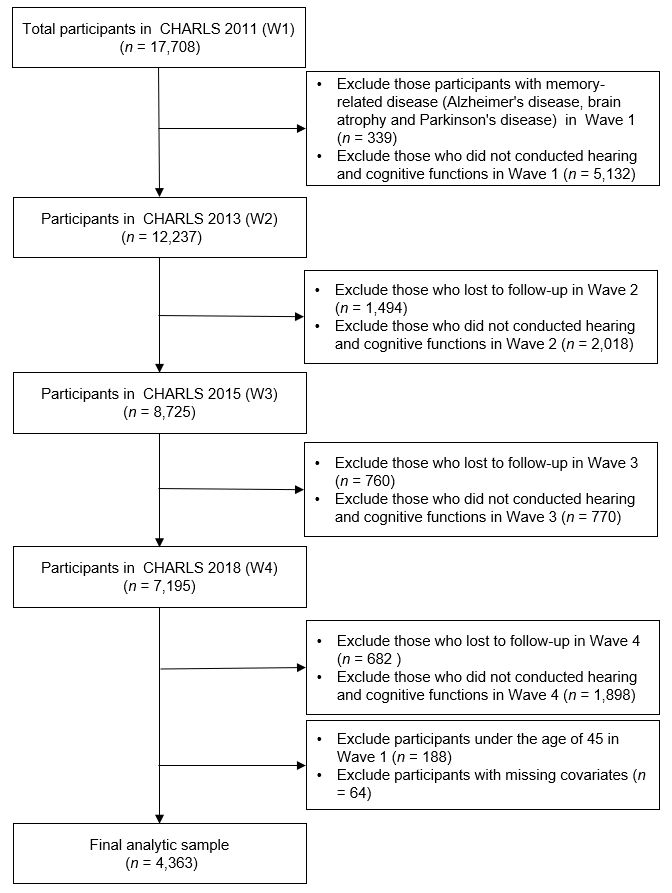


**Figure S1. Flow chart of the study participants.**

*Notes*: CHARLS = China Health and Retirement Longitudinal Study.


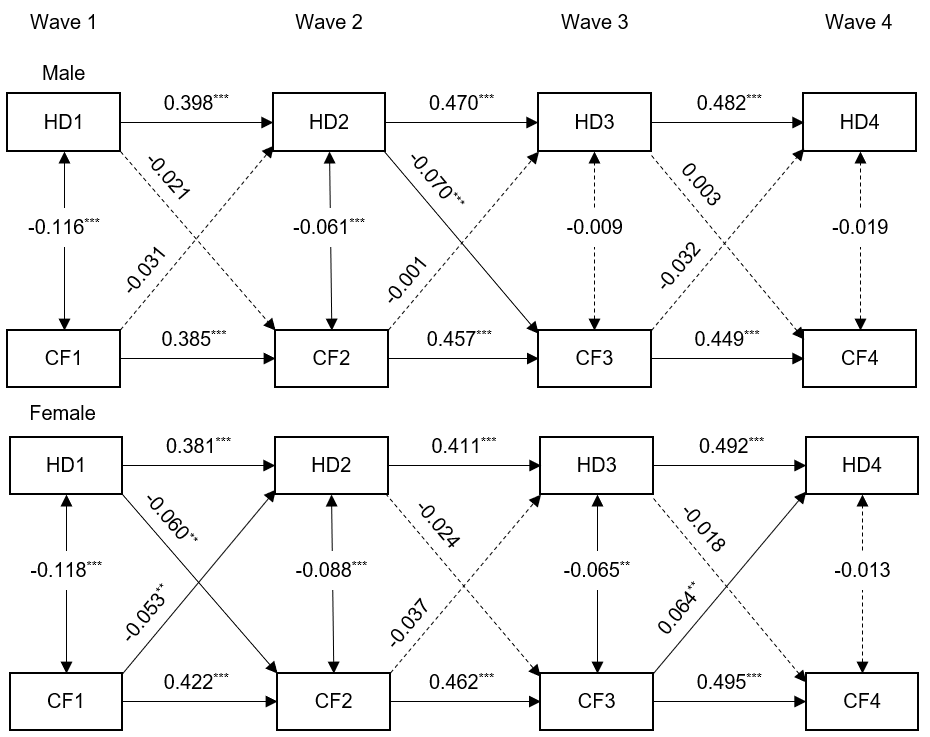


**Figure S2. Standardized coefficient estimates for the bidirectional longitudinal association between hearing difficulty and cognitive function by stratified by gender, CHARLS, 2011 - 2018.**

*Notes*: for simplicity, all covariates and residuals were estimated in the analysis but not shown in the diagram. Model adjusted for age, education level, marital status, smoking, drinking, hypertension, diabetes, and cardiovascular diseases. HD1, HD2, HD3, and HD4 = hearing difficulty at Wave 1, 2, 3, 4; CF1, CF2, CF3, and CF4 = cognitive function at Wave 1, 2, 3, 4. ^***^*p* < 0.001; ^**^*p* < 0.01; ^*^*p* < 0.05.


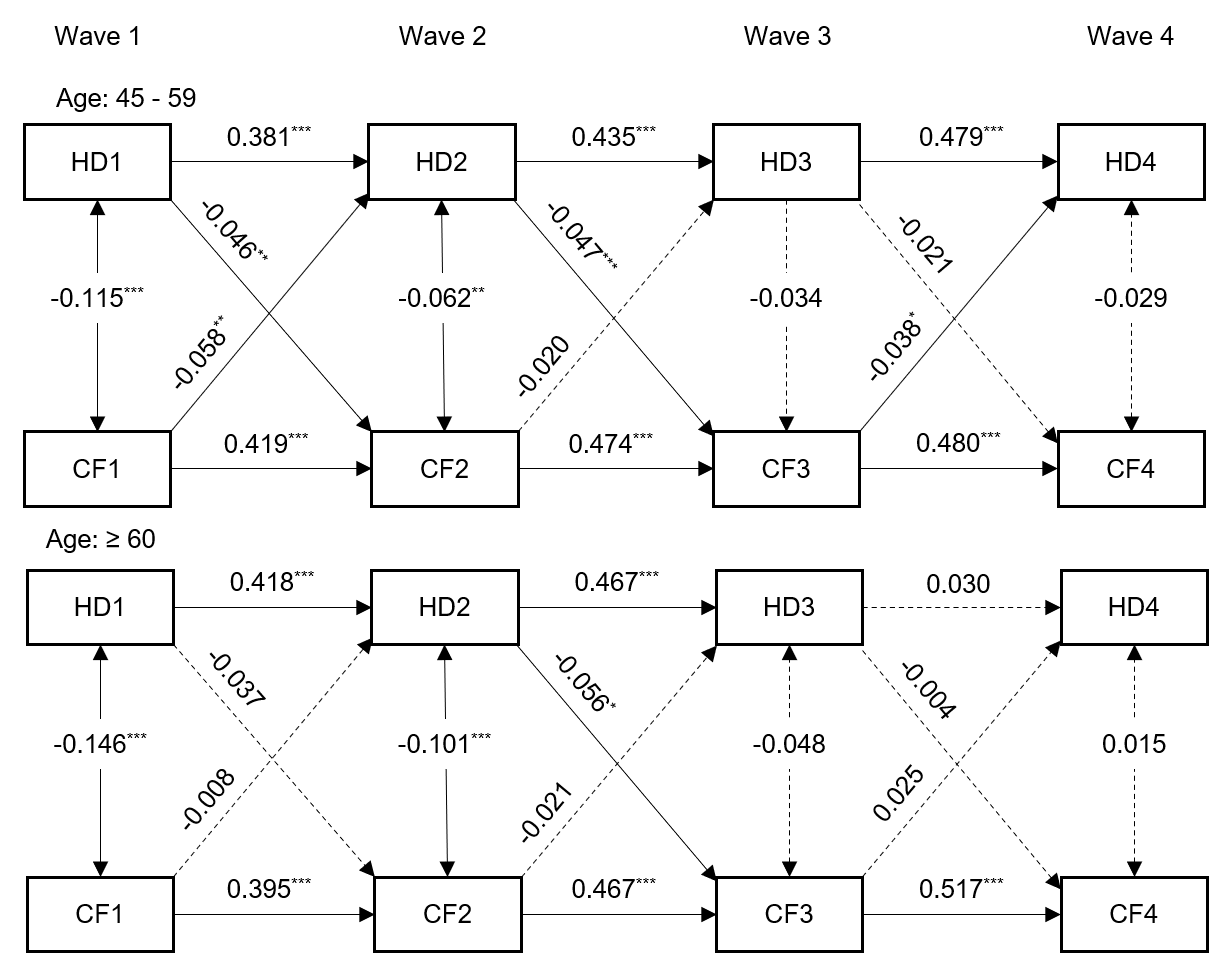


**Figure S3. Standardized coefficient estimates for the bidirectional longitudinal association between hearing difficulty and cognitive function by stratified by age, CHARLS, 2011 - 2018.**

*Notes*: for simplicity, all covariates and residuals were estimated in the analysis but not shown in the diagram. Model adjusted for gender, education level, marital status, smoking, drinking, hypertension, diabetes, and cardiovascular diseases. HD1, HD2, HD3, and HD4 = hearing difficulty at Wave 1, 2, 3, 4; CF1, CF2, CF3, and CF4 = cognitive function at Wave 1, 2, 3, 4. ^***^*p* < 0.001; ^**^*p* < 0.01; ^*^*p* < 0.05.


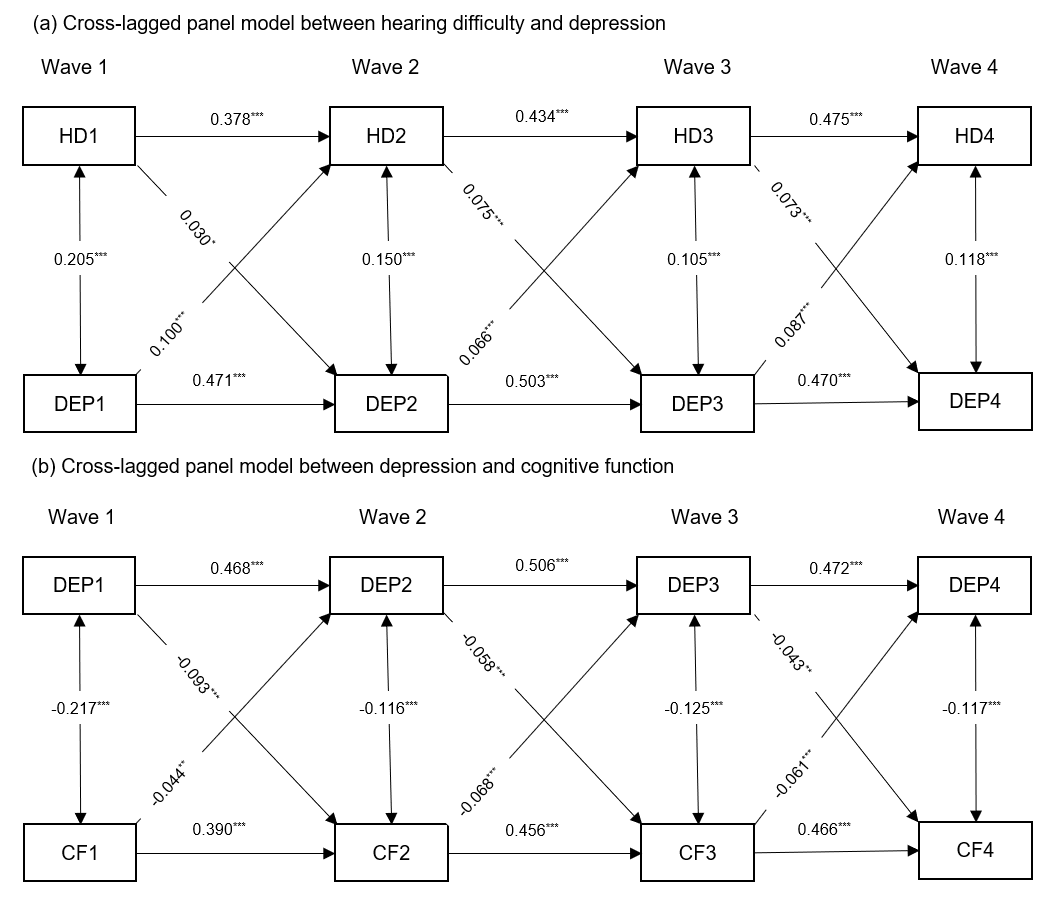


**Figure S4. Standardized coefficient estimates for the association between hearing difficulty,** **depressive symptoms, and cognitive function, CHARLS, 2011 - 2018.**

*Notes*: for simplicity, all covariates and residuals were estimated in the analysis but not shown in the diagram. Model adjusted for age, gender, education level, marital status, smoking, drinking, hypertension, diabetes, and cardiovascular diseases. HD1, HD2, HD3, and HD4 = hearing difficulty at Waves 1, 2, 3, 4; CF1, CF2, CF3, DEP1, DEP2, DEP3, and DEP4 = depressive symptoms at Wave 1, 2, 3, 4, and CF4 = cognitive function at Wave 1, 2, 3, 4. ^***^*p* < 0.001; ^**^*p* < 0.01; ^*^*p* < 0.05.

**Table S1.** Characteristics of the study population by inclusion and exclusion.

| Characteristics | | Population included  (*n* = 4,363) | Population excluded  (n =13,345) | *p* |
| --- | --- | --- | --- | --- |
| Age, years (mean ± SD) ^a^ | | 56.16 ± 7.75 | 59.27 ± 10.75 | < 0.001 |
| Sex, *n* (%) ^a^ | |  |  | < 0.001 |
|  | Male | 2,407 (55.17) | 6,071 (45.50) |  |
|  | Female | 1,956 (44.83) | 7,272 (54.50) |  |
| Level of education, *n* (%) ^a^ | |  |  | < 0.001 |
|  | Less than lower secondary | 3,555 (81.48) | 11,990 (89.95) |  |
|  | Upper secondary & vocational training or above | 808 (18.52) | 1,339 (10.05) |  |
| Marital status, *n* (%) ^a^ | |  |  | < 0.001 |
|  | Separated/divorced/widowed/never married | 296 (6.78) | 1,952 (14.64) |  |
|  | Married/partnered | 4,067 (93.22) | 11,381 (85.36) |  |
| Alcohol consumption, *n* (%) ^a^ | |  |  | < 0.001 |
|  | Never | 2,706 (62.02) | 9,079 (68.84) |  |
|  | Current | 1,657 (37.98) | 4,110 (31.16) |  |
| Smoking, *n* (%) ^a^ | |  |  | < 0.001 |
|  | Never | 2,501 (57.32) | 8,118 (61.52) |  |
|  | Former | 415 (9.51) | 1,654 (12.53) |  |
|  | Current | 1,447 (33.17) | 3,424 (25.95) |  |
| High blood pressure, *n* (%) ^a^ | |  |  | 0.004 |
|  | No | 3,303 (75.70) | 9,632 (73.49) |  |
|  | Yes | 1,060 (24.30) | 3,475 (26.51) |  |
| Diabetes, *n* (%) ^a^ | |  |  | 0.429 |
|  | No | 4,086 (93.65) | 12,260 (93.98) |  |
|  | Yes | 277 (6.35) | 785 (6.02) |  |
| Cardiovascular diseases, *n* (%) ^a^ | |  |  | 0.917 |
|  | No | 3,829 (87.76) | 11,508 (87.82) |  |
|  | Yes | 534 (12.24) | 1,596 (12.18) |  |

*Notes*:  ^a^ Data were missing for the following characteristics: age (n = 175), sex (n = 2), level of education (n = 16), marital status (n = 12), alcohol consumption (n=156), smoking (n=149), high blood pressure (n=238), Diabetes (n=300), cardiovascular diseases (n=241), tobacco use (n = 3,328), alcohol use (n = 87), chronic diseases (n = 67).

**Table S2.** Baseline characteristics of study participants according to hearing status and quartile score of cognitive function at Wave 4.

| Characteristics | | Total  (*n* = 4,363) | Hearing status | | | Cognitive function | | | | |
| --- | --- | --- | --- | --- | --- | --- | --- | --- | --- | --- |
|  |  |  | Without Hearing Difficulty (*n* = 1,495) | Hearing Difficulty  (*n* = 2,868) | *p* | Q1  (*n* = 1,189) | Q2  (*n* = 1,296) | Q3  (*n* = 1,035) | Q4  (*n* =843) | *p* |
| Age, years (mean ± SD) | | 56.16 ± 7.75 | 54.96 ± 7.41 | 56.78 ± 7.85 | < 0.001 | 58.91 ± 7.83 | 56.45 ± 7.54 | 54.81 ± 7.25 | 53.49 ± 7.22 | < 0.001 |
| Sex, *n* (%) | |  |  |  | 0.574 |  |  |  |  | < 0.001 |
|  | Male | 2,407 (55.17) | 816 (54.58) | 1,591 (55.47) |  | 667 (56.10) | 758 (58.49) | 582 (56.23) | 400 (47.45) |  |
|  | Female | 1,956 (44.83) | 679 (45.48) | 1,277 (44.53) |  | 522 (43.90) | 538 (41.51) | 453 (43.77) | 443 (52.55) |  |
| Level of education, *n* (%) | |  |  |  | < 0.001 |  |  |  |  | < 0.001 |
|  | Less than lower secondary | 3,555 (81.48) | 1,121 (74.98) | 2,434 (84.87) |  | 1,104 (92.85) | 1091 (84.18) | 794 (76.71) | 566 (67.14) |  |
|  | Upper secondary & vocational training or above | 808 (18.52) | 374 (25.02) | 434 (15.13) |  | 85 (7.15) | 205 (15.82) | 241 (23.29) | 277 (32.86) |  |
| Marital status, *n* (%) | |  |  |  | 0.957 |  |  |  |  | < 0.001 |
|  | Separated/divorced/widowed/never married | 296 (6.78) | 101 (6.76) | 195 (6.80) |  | 115 (9.67) | 86 (6.64) | 49 (4.73) | 46 (5.46) |  |
|  | Married/partnered | 4,067 (93.22) | 1,394 (93.24) | 2,673 (93.20) |  | 1,074 (90.33) | 1,210 (93.36) | 986(95.27) | 797 (94.54) |  |
| Alcohol consumption, *n* (%) | |  |  |  | 0.832 |  |  |  |  | 0.457 |
|  | Never | 2,706 (62.02) | 924 (61.81) | 1,782 (62.13) |  | 740 (62.24) | 781 (60.26) | 653 (63.09) | 532 (63.11) |  |
|  | Current | 1,657 (37.98) | 571 (38.19) | 1,086 (37.87) |  | 449 (37.76) | 515 (39.74) | 382 (36.91) | 311 (36.89) |  |
| Smoking, *n* (%) | |  |  |  | 0.303 |  |  |  |  | < 0.001 |
|  | Never | 2,501 (57.32) | 867 (57.99) | 1,634 (56.97) |  | 637 (53.57) | 705 (54.40) | 614 (59.32) | 545 (64.65) |  |
|  | Former | 415 (9.51) | 128 (8.56) | 287 (10.01) |  | 122 (10.26) | 121 (9.34) | 100 (9.66) | 72 (8.54) |  |
|  | Current | 1,447 (33.17) | 500 (33.44) | 947 (33.02) |  | 430 (36.16) | 470 (36.27) | 321 (31.01) | 226 (26.81) |  |
| High blood pressure, *n* (%) | |  |  |  | 0.051 |  |  |  |  | 0.161 |
|  | No | 3,303 (75.70) | 1,158 (77.46) | 2,145 (74.79) |  | 893 (75.11) | 972 (75.00) | 778 (75.17) | 660 (78.29) |  |
|  | Yes | 1,060 (24.30) | 337 (22.54) | 723 (25.21) |  | 296 (24.89) | 324 (25.00) | 257 (24.83) | 183 (21.71) |  |
| Diabetes, *n* (%) | |  |  |  | 0.520 |  |  |  |  | 0.382 |
|  | No | 4,086 (93.65) | 1,405 (93.98) | 2,681 (93.48) |  | 1,121 (94.28) | 1,188(91.67) | 982(94.88) | 795 (94.31) |  |
|  | Yes | 277 (6.35) | 90 (6.02) | 187 (6.52) |  | 68 (5.72) | 108(8.33) | 53(5.12) | 48 (5.69) |  |
| Cardiovascular diseases, *n* (%) | |  |  |  | < 0.001 |  |  |  |  | 0.088 |
|  | No | 3,829 (87.76) | 1,363 (91.17) | 2,466 (85.98) |  | 1,071 (90.08) | 1,116 (86.11) | 906 (87.54) | 736 (87.31) |  |
|  | Yes | 534 (12.24) | 132 (8.83) | 402 (14.02) |  | 118 (9.92) | 180 (13.89) | 129 (12.46) | 107 (12.69) |  |
| HD at Wave 1 (mean ± SD) | | 3.39 ± 0.92 | 2.96 ± 0.85 | 3.62 ± 0.87 | < 0.001 | 3.49 ± 0.92 | 3.40 ± 0.93 | 3.35 ± 0.93 | 3.30 ± 0.86 | < 0.001 |
| CF at Wave 1 (mean ± SD) | | 16.55 ± 4.00 | 17.10 ± 4.04 | 16.27 ± 3.95 | < 0.001 | 14.43 ± 4.10 | 16.27 ± 3.59 | 17.47 ± 3.39 | 18.85 ± 3.53 | < 0.001 |

*Notes*: SD = standard deviation. HD = hearing difficulty; CF = cognitive function.

**Table S3. Results of correlation coefficient matrix of study variable across different waves in CHARLS (*n* = 4,363).**

| Variable | 1 | 2 | 3 | 4 | 5 | 6 | 7 | 8 | 9 | 10 | 11 | 12 |
| --- | --- | --- | --- | --- | --- | --- | --- | --- | --- | --- | --- | --- |
| 1 T1 hearing status | 1 |  |  |  |  |  |  |  |  |  |  |  |
| 2 T1 depressive symptoms | 0.216^***^ | 1 |  |  |  |  |  |  |  |  |  |  |
| 3 T1 cognitive function | -0.139^***^ | -0.241^***^ | 1 |  |  |  |  |  |  |  |  |  |
| 4 T2 hearing status | 0.411^***^ | 0.189^***^ | -0.113^***^ | 1 |  |  |  |  |  |  |  |  |
| 5 T2 depressive symptoms | 0.137^***^ | 0.496^***^ | -0.162^***^ | 0.222^***^ | 1 |  |  |  |  |  |  |  |
| 6 T2 cognitive function | -0.115^***^ | -0.209^***^ | 0.445^***^ | -0.142^***^ | -0.210^***^ | 1 |  |  |  |  |  |  |
| 7 T3 hearing status | 0.411^***^ | 0.177^***^ | -0.111^***^ | 0.460^***^ | 0.168^***^ | -0.100^***^ | 1 |  |  |  |  |  |
| 8 T3 depressive symptoms | 0.134^***^ | 0.470^***^ | -0.173^***^ | 0.192^***^ | 0.535^***^ | -0.184^***^ | 0.202^***^ | 1 |  |  |  |  |
| 9 T3 cognitive function | -0.126^***^ | -0.179^***^ | 0.465^***^ | -0.136^***^ | -0.162^***^ | 0.519^***^ | -0.119^***^ | -0.210^***^ | 1 |  |  |  |
| 10 T4 hearing status | 0.390^***^ | 0.178^***^ | -0.100^**^ | 0.432^***^ | 0.191^***^ | -0.085^***^ | 0.505^***^ | 0.188^***^ | -0.126^***^ | 1 |  |  |
| 11 T4 depressive symptoms | 0.146^***^ | 0.419*^**^ | -0.165^***^ | 0.170^***^ | 0.496^***^ | -0.176^***^ | 0.174^***^ | 0.505^***^ | -0.169^***^ | 0.214^***^ | 1 |  |
| 12 T4 cognitive function | -0.085^***^ | -0.150^***^ | 0.425^***^ | -0.085^***^ | -0.141^***^ | 0.470^***^ | -0.091^***^ | -0.151^***^ | 0.541^***^ | -0.106^***^ | -0.192^***^ | 1 |

*Notes*: ^***^*p* < 0.001; ^**^*p* < 0.01; ^*^*p* < 0.05.

**Table S4. Model fits and comparisons for cross-lagged panel models.**

| Models | Model fits | | | | Model comparisons | | | | | |
| --- | --- | --- | --- | --- | --- | --- | --- | --- | --- | --- |
|  | χ2 | *df* | CFI | RMSEA [90% CI] | Pairs | Δχ2 | Δ *df* | *p* | ΔCFI | ΔRMSEA |
| Model 4 | 1485.956 | 12 | 0.849 | 0.168 [0.161, 0.175] |  |  |  |  |  |  |
| Model 5 | 1492.438 | 16 | 0.849 | 0.145 [0.139, 0.152] | Model 5 - Model 4 | 6.482 | 4 | 0.166 | 0.000 | -0.023 |
| Model 6 | 1562.102 | 16 | 0.842 | 0.149 [0.143, 0.155] | Model 6 - Model 4 | 76.146 | 4 | < 0.001 | -0.007 | -0.019 |
| Model 7 | 1493.454 | 14 | 0.849 | 0.156 [0.149, 0.162] | Model 7 - Model 4 | 7.498 | 2 | 0.024 | 0.000 | -0.012 |
| Model 8 | 1574.976 | 22 | 0.841 | 0.127 [0.122, 0.133] | Model 8 - Model 4 | 89.02 | 10 | < 0.001 | -0.008 | -0.041 |

*Notes*: Model 4 = unconstrained baseline model; Model 5 = model with all cross-lagged paths fixed to be time-invariant; Model 6 = model with all stability paths fixed to be time-invariant; Model 7 = model with all Wave 2–Wave 4 correlated changes fixed to be time-invariant; Model 8 = model with all cross-lagged paths, all stability paths, and all Wave 2 - Wave 4 correlated changes fixed to be time-invariant; χ2 = chi-square value; CFI = comparative fit index; RMSEA = root mean square error of approximation; 90% CI = 90% confidence interval; Δ = change in parameter.

**Table S5. Model fit indices and standardized path coefficients for cross-lagged models by stratified by gender, CHARLS (*n* = 4,363), 2011 - 2018.**

| Paths | Male (*n* = 2,407) | | Female (*n* = 1,956) | |
| --- | --- | --- | --- | --- |
|  | β | SE | β | SE |
| Autoregressive paths | | | | |
| HD1 ⭢ HD2 | 0.398^***^ | 0.017 | 0.381^***^ | 0.020 |
| HD2 ⭢ HD3 | 0.470^***^ | 0.016 | 0.411^***^ | 0.019 |
| HD3 ⭢ HD4 | 0.482^***^ | 0.016 | 0.492^***^ | 0.017 |
| CF1 ⭢ CF2 | 0.385^***^ | 0.018 | 0.422^***^ | 0.019 |
| CF2 ⭢ CF3 | 0.457^***^ | 0.016 | 0.462^***^ | 0.018 |
| CF3 ⭢ CF4 | 0.449^***^ | 0.016 | 0.495^***^ | 0.017 |
| Cross-lagged paths | | | | |
| HD1 ⭢ CF2 | -0.021 | 0.019 | -0.060^**^ | 0.020 |
| CF1 ⭢ HD2 | -0.031 | 0.019 | -0.053^*^ | 0.022 |
| HD2 ⭢ CF3 | -0.070^***^ | 0.017 | -0.024 | 0.019 |
| CF2 ⭢ HD3 | -0.001 | 0.018 | -0.037 | 0.022 |
| HD3 ⭢ CF4 | 0.003 | 0.017 | -0.018 | 0.018 |
| CF3⭢ HD4 | -0.032 | 0.018 | -0.064^**^ | 0.021 |
| Residual correlations | | | | |
| HD1 with CF1 | -0.116^***^ | 0.020 | -0.118^***^ | 0.022 |
| HD2 with CF2 | -0.061^**^ | 0.020 | -0.088^***^ | 0.022 |
| HD3 with CF3 | -0.009 | 0.020 | -0.065^**^ | 0.023 |
| HD4 with CF4 | -0.019 | 0.020 | -0.013 | 0.023 |
| Model fit indices | | | | |
| CFI | 0.854 | | 0.847 | |
| TLI | -0.220 | | -0.271 | |
| SRMR | 0.040 | | 0.043 | |
| RMSEA | 0.164 | | 0.171 | |

*Notes*: Model adjusted for age, education level, marital status, smoking, drinking, hypertension, diabetes, and cardiovascular diseases. HD1, HD2, HD3, and HD4 = hearing difficulty at Wave 1, 2, 3, 4; CF1, CF2, CF3, and CF4 = cognitive function at Wave 1, 2, 3, 4. ^***^*p* < 0.001; ^**^*p* < 0.01; ^*^*p* < 0.05.

**Table S6. Model fit indices and standardized path coefficients for cross-lagged models by stratified by education, CHARLS (*n* = 4,363), 2011 - 2018.**

| Paths | Low (*n* = 3,555) | | High (*n* = 808) | |
| --- | --- | --- | --- | --- |
|  | β | SE | β | SE |
| Autoregressive paths | | | | |
| HD1 ⭢ HD2 | 0.376^***^ | 0.015 | 0.471^***^ | 0.028 |
| HD2 ⭢ HD3 | 0.425^***^ | 0.014 | 0.529^***^ | 0.026 |
| HD3 ⭢ HD4 | 0.482^***^ | 0.013 | 0.509^***^ | 0.026 |
| CF1 ⭢ CF2 | 0.418^***^ | 0.014 | 0.341^***^ | 0.031 |
| CF2 ⭢ CF3 | 0.470^***^ | 0.013 | 0.410^***^ | 0.029 |
| CF3 ⭢ CF4 | 0.483^***^ | 0.013 | 0.394^***^ | 0.030 |
| Cross-lagged paths | | | | |
| HD1 ⭢ CF2 | -0.031^*^ | 0.015 | -0.077^*^ | 0.032 |
| CF1 ⭢ HD2 | -0.038^*^ | 0.016 | -0.059 | 0.031 |
| HD2 ⭢ CF3 | -0.041^**^ | 0.015 | -0.089^**^ | 0.031 |
| CF2 ⭢ HD3 | -0.013 | 0.015 | -0.025 | 0.031 |
| HD3 ⭢ CF4 | -0.005 | 0.014 | -0.030 | 0.031 |
| CF3⭢ HD4 | -0.048^**^ | 0.013 | -0.039 | 0.031 |
| Residual correlations | | | | |
| HD1 with CF1 | -0.125^***^ | 0.017 | -0.080^*^ | 0.035 |
| HD2 with CF2 | -0.073^***^ | 0.017 | -0.085^*^ | 0.035 |
| HD3 with CF3 | -0.040^*^ | 0.017 | -0.008 | 0.035 |
| HD4 with CF4 | -0.029 | 0.017 | 0.036 | 0.035 |
| Model fit indices | | | | |
| CFI | 0.828 | | 0.837 | |
| TLI | -0.437 | | -0.360 | |
| SRMR | 0.043 | | 0.041 | |
| RMSEA | 0.167 | | 0.168 | |

*Notes*: Model adjusted for age, gender, marital status, smoking, drinking, hypertension, diabetes, and cardiovascular diseases. HD1, HD2, HD3, and HD4 = hearing difficulty at Wave 1, 2, 3, 4; CF1, CF2, CF3, and CF4 = cognitive function at Wave 1, 2, 3, 4. ^***^*p* < 0.001; ^**^*p* < 0.01; ^*^*p* < 0.05.

**Table S7.** Model fit indices and standardized path coefficients for cross-lagged models by stratified by age, CHARLS (*n* = 4,363), 2011 - 2018.

| Paths | 45 – 59 (*n* = 2,916) | | ≥60 (*n* = 1,447) | |
| --- | --- | --- | --- | --- |
|  | β | SE | β | SE |
| Autoregressive paths | | | | |
| HD1 ⭢ HD2 | 0.381^***^ | 0.016 | 0.418^***^ | 0.022 |
| HD2 ⭢ HD3 | 0.435^***^ | 0.015 | 0.467^***^ | 0.021 |
| HD3 ⭢ HD4 | 0.479^***^ | 0.014 | 0.030 | 0.027 |
| CF1 ⭢ CF2 | 0.419^***^ | 0.015 | 0.395^***^ | 0.022 |
| CF2 ⭢ CF3 | 0.474^***^ | 0.015 | 0.467^***^ | 0.021 |
| CF3 ⭢ CF4 | 0.480^***^ | 0.015 | 0.517^***^ | 0.020 |
| Cross-lagged paths | | | | |
| HD1 ⭢ CF2 | -0.046^**^ | 0.016 | -0.037 | 0.024 |
| CF1 ⭢ HD2 | -0.058^**^ | 0.018 | -0.008 | 0.024 |
| HD2 ⭢ CF3 | -0.047^**^ | 0.016 | -0.056^*^ | 0.023 |
| CF2 ⭢ HD3 | -0.020 | 0.017 | -0.021 | 0.024 |
| HD3 ⭢ CF4 | -0.021 | 0.016 | 0.004 | 0.022 |
| CF3⭢ HD4 | -0.038^*^ | 0.017 | 0.025 | 0.027 |
| Residual correlations | | | | |
| HD1 with CF1 | -0.115^***^ | 0.018 | -0.146^***^ | 0.026 |
| HD2 with CF2 | -0.062^**^ | 0.018 | -0.101^***^ | 0.026 |
| HD3 with CF3 | -0.034 | 0.018 | -0.048 | 0.026 |
| HD4 with CF4 | -0.029 | 0.019 | 0.015 | 0.026 |
| Model fit indices | | | | |
| CFI | 0.836 | | 0.844 | |
| TLI | -0.365 | | -0.303 | |
| SRMR | 0.043 | | 0.036 | |
| RMSEA | 0.168 | | 0.148 | |

*Notes*: Model adjusted for gender, education level, marital status, smoking, drinking, hypertension, diabetes, and cardiovascular diseases. HD1, HD2, HD3, and HD4 = hearing difficulty at Wave 1, 2, 3, 4; CF1, CF2, CF3, and CF4 = cognitive function at Wave 1, 2, 3, 4. ^***^*p* < 0.001; ^**^*p* < 0.01; ^*^*p* < 0.05.

**Table S8. Model fits and comparisons for random-intercept cross-lagged panel models.**

| Models | Model fits | | | | Model comparisons | | | | | |
| --- | --- | --- | --- | --- | --- | --- | --- | --- | --- | --- |
|  | χ2 | *df* | CFI | RMSEA [90% CI] | Pairs | Δχ2 | Δ *df* | *p* | ΔCFI | ΔRMSEA |
| Model 4 | 31.850 | 7 | 0.997 | 0.029 [0.019, 0.039] |  |  |  |  |  |  |
| Model 5 | 37.838 | 11 | 0.997 | 0.024 [0.016, 0.032] | Model 5 - Model 4 | 5.988 | 4 | 0.200 | 0.000 | -0.005 |
| Model 6 | 95.738 | 11 | 0.991 | 0.042 [0.035, 0.050] | Model 6 - Model 4 | 63.888 | 4 | < 0.001 | -0.006 | 0.013 |
| Model 7 | 39.807 | 9 | 0.997 | 0.028 [0.019, 0.037] | Model 7 - Model 4 | 7.957 | 2 | 0.019 | 0.000 | -0.001 |
| Model 8 | 113.292 | 17 | 0.990 | 0.036 [0.030, 0.042] | Model 8 - Model 4 | 81.442 | 10 | < 0.001 | -0.007 | 0.007 |

*Notes*: Model 4 = unconstrained baseline model; Model 5 = model with all cross-lagged paths fixed to be time-invariant; Model 6 = model with all stability paths fixed to be time-invariant; Model 7 = model with all Wave 2–Wave 4 correlated changes fixed to be time-invariant; Model 8 = model with all cross-lagged paths, all stability paths, and all Wave 2–Wave 4 correlated changes fixed to be time-invariant; χ2 = chi-square value; CFI = comparative fit index; RMSEA = root mean square error of approximation; 90% CI = 90% confidence interval; Δ = change in parameter.

**Table S9. Model fit indices and standardized path coefficients for random intercept cross-lagged models between hearing difficulty and cognitive function, CHARLS (*n* = 4,363), 2011 - 2018.**

| Paths | Model 1 | | Model 2 | | Model 3 | | Model 4 | | Model 5 | |
| --- | --- | --- | --- | --- | --- | --- | --- | --- | --- | --- |
|  | β | SE | β | SE | β | SE | β | SE | β | SE |
| Autoregressive paths | | | | | | | | | | |
| HD1 ⭢ HD2 | 0.030 | 0.022 | 0.032 | 0.022 | 0.030 | 0.022 | 0.031 | 0.022 | 0.028 | 0.022 |
| HD2 ⭢ HD3 | 0.103^***^ | 0.022 | 0.103^***^ | 0.022 | 0.103^***^ | 0.022 | 0.105^***^ | 0.022 | 0.102^***^ | 0.022 |
| HD3 ⭢ HD4 | 0.154^***^ | 0.021 | 0.155^***^ | 0.021 | 0.156^***^ | 0.021 | 0.157^***^ | 0.021 | 0.158^***^ | 0.021 |
| CF1 ⭢ CF2 | -0.098^***^ | 0.028 | -0.054^*^ | 0.025 | -0.054^*^ | 0.025 | -0.053^*^ | 0.025 | -0.053^*^ | 0.025 |
| CF2 ⭢ CF3 | 0.027 | 0.026 | 0.028 | 0.025 | 0.029 | 0.025 | 0.029 | 0.025 | 0.037 | 0.025 |
| CF3 ⭢ CF4 | 0.203^***^ | 0.019 | 0.156^***^ | 0.019 | 0.157^***^ | 0.019 | 0.156^***^ | 0.020 | 0.155^***^ | 0.019 |
| Cross-lagged paths | | | | | | | | | | |
| HD1 ⭢ CF2 | 0.006 | 0.027 | -0.019 | 0.025 | -0.019 | 0.025 | -0.016 | 0.025 | -0.041^**^ | 0.014 |
| CF1 ⭢ HD2 | -0.015 | 0.024 | -0.022 | 0.023 | -0.021 | 0.023 | -0.021 | 0.023 | -0.025 | 0.012 |
| HD2 ⭢ CF3 | -0.095^***^ | 0.027 | -0.091^**^ | 0.027 | -0.089^**^ | 0.027 | -0.089^**^ | 0.027 | -0.043^**^ | 0.015 |
| CF2 ⭢ HD3 | -0.021 | 0.026 | -0.026 | 0.026 | -0.025 | 0.026 | -0.023^*^ | 0.024 | -0.025 | 0.013 |
| HD3 ⭢ CF4 | -0.029 | 0.020 | -0.018 | 0.021 | -0.018 | 0.027 | -0.018 | 0.021 | -0.032^**^ | 0.011 |
| CF3⭢ HD4 | -0.077^**^ | 0.024 | -0.061^*^ | 0.024 | -0.060^*^ | 0.024 | -0.062^*^ | 0.024 | -0.026 | 0.013 |
| Residual correlations | | | | | | | | | | |
| HD1 with CF1 | 0.007 | 0.031 | -0.013 | 0.030 | -0.013 | 0.030 | -0.013 | 0.030 | -0.039 | 0.021 |
| HD2 with CF2 | -0.117^***^ | 0.027 | -0.124^***^ | 0.025 | -0.123^***^ | 0.025 | -0.121^***^ | 0.025 | -0.109^***^ | 0.023 |
| HD3 with CF3 | -0.053^*^ | 0.027 | -0.045 | 0.027 | -0.044 | 0.027 | -0.044 | 0.027 | -0.024 | 0.021 |
| HD4 with CF4 | -0.054^**^ | 0.019 | -0.037 | 0.019 | -0.037 | 0.019 | -0.040^*^ | 0.019 | -0.034 | 0.018 |
| RI_HD with RI_CF | -0.178^***^ | 0.029 | -0.076^*^ | 0.032 | -0.098^**^ | 0.032 | -0.102^**^ | 0.032 | -0.123^***^ | 0.028 |
| Model fit indices | | | | | | | | | | |
| CFI | 0.996 | | 0.997 | | 0.997 | | 0.997 | | 0.997 | |
| TLI | 0.984 | | 0.978 | | 0.969 | | 0.961 | | 0.973 | |
| SRMR | 0.021 | | 0.011 | | 0.009 | | 0.008 | | 0.008 | |
| RMSEA | 0.034 | | 0.029 | | 0.029 | | 0.029 | | 0.024 | |

*Notes*: Model 1 was built without any adjustments; Model 2 is adjusted for age, sex, education level, marital status; Model 3 is adjusted for the covariates in model 2 plus smoking, drinking; Model 4 is adjusted for the covariates in model 3 plus hypertension, diabetes, and cardiovascular diseases; Model 5 = model 4 with all cross-lagged paths fixed to be time-invariant; ^***^*p* < 0.001; ^**^*p* < 0.01; ^*^*p* < 0.05. HD1, HD2, HD3, and HD4 = hearing difficulty at Wave 1, 2, 3, 4; CF1, CF2, CF3, and CF4 = cognitive function at Wave 1, 2, 3, 4; RI = random intercepts.

**Table S10. Model fit indices and standardized path coefficients for longitudinal mediation models, CHARLS (*n* = 4,363), 2011-2018.**

| Paths | β  SE | Boot LL CI | Boot UL CI |
| --- | --- | --- | --- |
| Autoregressive paths | | | |
| HD1 ⭢ HD2 | 0.377^***^ | 0.352 | 0.399 |
| HD2 ⭢ HD3 | 0.432^***^ | 0.410 | 0.454 |
| HD3 ⭢ HD4 | 0.474^***^ | 0.450 | 0.496 |
| DEP1 ⭢ DEP2 | 0.462^***^ | 0.437 | 0.486 |
| DEP2 ⭢ DEP3 | 0.492^***^ | 0.467 | 0.515 |
| DEP3 ⭢ DEP4 | 0.458^***^ | 0.434 | 0.483 |
| CF1 ⭢ CF2 | 0.389^***^ | 0.366 | 0.412 |
| CF2 ⭢ CF3 | 0.452^***^ | 0.431 | 0.473 |
| CF3 ⭢ CF4 | 0.466^***^ | 0.445 | 0.487 |
| Residual correlations | | | |
| HD1 with DEP1 | 0.183^***^ | 0.160 | 0.206 |
| DEP1 with CF1 | -0.197^***^ | -0.220 | -0.173 |
| HD2 with DEP2 | 0.143^***^ | 0.118 | 0.169 |
| DEP2 with CF2 | -0.106^***^ | -0.131 | -0.080 |
| HD3 with DEP3 | 0.101^***^ | 0.077 | 0.125 |
| DEP3 with CF3 | -0.119^***^ | -0.146 | -0.095 |
| HD4 with DEP4 | 0.117^***^ | 0.093 | 0.142 |
| DEP4 with CF4 | -0.119^***^ | -0.143 | -0.093 |
| Cross-lagged paths | | | |
| HD1 ⭢ CF2 | -0.023 | -0.045 | -0.001 |
| DEP1 ⭢ CF2 | -0.088^***^ | -0.112 | -0.063 |
| HD1 ⭢ DEP2 | 0.027^*^ | 0.005 | 0.050 |
| CF1⭢ DEP2 | -0.042^**^ | -0.065 | -0.018 |
| DEP1 ⭢ HD2 | 0.096^***^ | 0.072 | 0.119 |
| CF1 ⭢ HD2 | -0.022 | -0.047 | 0.002 |
| HD2 ⭢ CF3 | -0.024 | -0.048 | -0.002 |
| DEP2 ⭢ CF3 | -0.049^***^ | -0.070 | -0.027 |
| HD2 ⭢ DEP3 | 0.070^***^ | 0.050 | 0.092 |
| CF2⭢ DEP3 | -0.063^***^ | -0.086 | -0.040 |
| DEP2 ⭢ HD3 | 0.062^***^ | 0.040 | 0.085 |
| CF2⭢ HD3 | 0.010 | -0.015 | 0.035 |
| HD3 ⭢ CF4 | -0.010 | -0.032 | 0.014 |
| DEP3 ⭢ CF4 | -0.046^**^ | -0.068 | -0.023 |
| HD3 ⭢ DEP4 | 0.070^***^ | 0.050 | 0.091 |
| CF3⭢ DEP4 | -0.058^***^ | -0.081 | -0.034 |
| DEP3 ⭢ HD4 | 0.083^***^ | 0.062 | 0.105 |
| CF3⭢ HD4 | -0.041^*^ | -0.068 | -0.015 |
| HD1 ⭢ CF3 | -0.036^*^ | -0.058 | -0.012 |
| CF1 ⭢ HD3 | -0.038^*^ | -0.062 | -0.013 |
| HD2 ⭢ CF4 | 0.024 | 0.001 | 0.049 |
| CF2 ⭢ HD4 | 0.022 | -0.004 | 0.048 |
| Indirect effect | | | |
| HD1 ⭢ DEP2⭢ CF3 | -0.001 | -0.003 | 0.000 |
| CF1⭢ DEP2 ⭢ HD3 | -0.003^*^ | -0.005 | -0.001 |
| HD2 ⭢ DEP3⭢ CF4 | -0.003^**^ | -0.005 | -0.002 |
| CF2⭢ DEP3 ⭢ HD4 | -0.005^***^ | -0.008 | -0.003 |
| Model Fits | | | |
| CFI | 0.858 | | |
| TLI | -0.013 | | |
| SRMR | 0.043 | | |
| RMSEA | 0.141 | | |

*Notes*: Adjusted for the covariates including age, gender, education level, marital status, smoking, drinking, hypertension, diabetes, and cardiovascular diseases. HD1, HD2, HD3, and HD4 = hearing difficulty at Wave 1, 2, 3, 4; CF1, CF2, CF3, and CF4 = cognitive function at Wave 1, 2, 3, 4; DEP1, DEP 2, DEP 3, and DEP 4 = depressive symptoms at Wave 1, 2, 3, 4. ^***^*p* < 0.001; ^**^*p* < 0.01; ^*^*p* < 0.05. LLCI = lower limit confidence interval; ULCI = Upper limit confidence interval.
